# Evaluating the real-world implementation of the Family Nurse Partnership in England: protocol for a data linkage study

**DOI:** 10.1136/bmjopen-2020-038530

**Published:** 2020-05-18

**Authors:** Francesca L Cavallaro, Ruth Gilbert, Linda Wijlaars, Eilis Kennedy, Ailsa Swarbrick, Jan van der Meulen, Katie Harron

**Affiliations:** 1 Great Ormond Street Institute of Child Health, University College London, London, UK; 2 Children, Young Adults and Families Directorate, Tavistock and Portman NHS Foundation Trust, London, UK; 3 Family Nurse Partnership National Unit, Tavistock and Portman NHS Foundation Trust, London, UK; 4 Department of Health Services Research and Policy, London School of Hygiene & Tropical Medicine, London, UK

**Keywords:** community child health, child protection, public health, health informatics

## Abstract

**Introduction:**

Almost 20 000 babies are born to teenage mothers each year in England, with poorer outcomes for mothers and babies than among older mothers. A nurse home visitation programme in the USA was found to improve a wide range of outcomes for young mothers and their children. However, a randomised controlled trial in England found no effect on short-term primary outcomes, although cognitive development up to age 2 showed improvement. Our study will use linked routinely collected health, education and social care data to evaluate the real-world effects of the Family Nurse Partnership (FNP) on child outcomes up to age 7, with a focus on identifying whether the FNP works better for particular groups of families, thereby informing programme targeting and resource allocation.

**Methods and analysis:**

We will construct a retrospective cohort of all women aged 13–24 years giving birth in English NHS hospitals between 2010 and 2017, linking information on mothers and children from FNP programme data, Hospital Episodes Statistics and the National Pupil Database. To assess the effectiveness of FNP, we will compare outcomes for eligible mothers ever and never enrolled in FNP, and their children, using two analysis strategies to adjust for measured confounding: propensity score matching and analyses adjusting for maternal characteristics up to enrolment/28 weeks gestation. Outcomes of interest include early childhood development, childhood unplanned hospital admissions for injury or maltreatment-related diagnoses and children in care. Subgroup analyses will determine whether the effect of FNP varied according to maternal characteristics (eg, age and education).

**Ethics and dissemination:**

The Nottingham Research Ethics Committee approved this study. Mothers participating in FNP were supportive of our planned research. Results will inform policy-makers for targeting home visiting programmes. Methodological findings on the accuracy and reliability of cross-sectoral data linkage will be of interest to researchers.

**Strengths and limitations of this study**

- This study will complement results from the Building Blocks trial in England, by evaluating the real-world effect of the Family Nurse Partnership (FNP) on a range of health, educational and social care outcomes up to age 7 years.
- The study cohort comprises joint trajectories for mothers and babies captured in Hospital Episode Statistics (HES; containing >97% births in England and information on subsequent healthcare use), with linked information from FNP programme data and the National Pupil Database (NPD; containing information on children’s and mothers’ educational attainment and social care).
- Evaluating outcomes for up to 31 000 FNP families and up to 1 million controls will provide statistical power to detect small differences, differences in rare outcomes and to perform subgroup analyses to determine whether the FNP is more effective in particular groups of families.
- No unique identifiers exist between HES and NPD databases, and linkage may be incomplete.
- Findings will inform the adaptation of the FNP and targeting of support for teenage and young mothers to specific subgroups.

## Introduction

Approximately 3% of babies each year (19 000 in 2018) are born to mothers aged below 20 in England and Wales.^1^ Teenage mothers are more likely to experience adversity and have rapid repeat pregnancies, and are less likely to be engaged with education and employment compared with older mothers.^2–5^ For their children, young maternal age is associated with higher incidence of preterm birth and low birth weight^6 7^ and a greater risk of child maltreatment and associated adverse long-term consequences including poorer physical, social, emotional and cognitive outcomes.^8–10^ These adverse maternal and child outcomes of teenage pregnancy, due to social adversity, disruption to education and employment, and child-rearing practices, are of major importance to public health research.^11 12^

The Family Nurse Partnership (FNP) is an intensive home visiting programme that aims to improve outcomes of pregnancies among vulnerable young mothers. Participants receive up to 64 home visits from early pregnancy until the child’s second birthday. Specifically, the FNP aims to improve birth outcomes, optimise child health and development and promote economic self-sufficiency among vulnerable mothers.^13^ Three randomised controlled trials in the USA found evidence of benefit on a number of maternal and child outcomes, including reductions in child maltreatment, child emergency department visits and subsequent births, as well as some improvements in child developmental outcomes and longer subsequent birth intervals.^14–18^ Effects on hospital visits for injury or maltreatment, children’s educational achievements and maternal educational qualifications were less consistent. A trial in the Netherlands found reduced child maltreatment reports by age 2,^19^ and a trial of a FNP-based model in Germany reported improved child development among high-risk women only, although no difference in subsequent births within 2 years.^20^ Non-randomised studies have reported reduced child maltreatment,^21^ infant death^22^ and subsequent births^23^ among FNP participants compared with controls, as well as different patterns of emergency room visits.^24^

Following evidence from the US trials, FNP was introduced in the UK in 2007. The FNP was rolled out via partnerships between local authorities (LAs) and healthcare organisations; to date, more than 130 LAs have implemented the programme. Teenage and young mothers who do not participate in the FNP receive usual care, consisting in comprehensive maternity care and at least five home visits by a health visitor, as well as a varying range of additional services;^25^ substantial variation in usual care exists between LAs.^26^ In England, the FNP was initially offered to pregnant teenagers aged under 20 years up to 28 weeks gestation, but since 2017, selected sites have expanded to include women up to 24 years with specific vulnerability criteria, and/or have implemented some personalisation of the programme.

Following trials in other countries, the Building Blocks trial randomised 1618 families in England between 2009 and 2010 and showed no benefit of FNP on primary outcomes up to the child’s second birthday (smoking in late pregnancy, birth weight, subsequent pregnancy or child A&E and hospital attendances).^27^ However, the interpretation of trial results was debated due to the choice of maternally reported early childhood development as a secondary outcome, for which a positive effect was observed, and strong support locally for the value of FNP on parent-child relationships.^28–30^ Follow-up has been extended up to the child’s seventh birthday, with a focus on child maltreatment.^31^

Evaluating complex interventions such as the FNP requires an understanding of the context in which they are delivered as well as the mechanisms of change, in order to investigate and interpret any differences in effects across contexts and populations.^32 33^ Indeed, differences in the comparator of usual care may explain differences in results between trials in the USA, England and the Netherlands; these may also vary over time and between LAs in England.^26 28^ Furthermore, evidence from the US trials suggests that the youngest, most disadvantaged mothers are likely to benefit most from FNP.^28^ Adequately powered subgroup analyses are needed to examine whether some groups of families benefited from FNP more than others. In addition, constrained conditions under which trials are conducted often do not match the complexity of real-world implementation of programmes.^34^

Linkage of existing administrative records provides a cost-efficient means of evaluating services as they are implemented in the real world, by bringing together data from different sectors on a range of outcomes. They also allow for a sufficiently large sample size for subgroup analyses. Our population-based study will use longitudinal observational data to evaluate effects on outcomes of mothers and children up to age 7 among eligible families and generate evidence on the factors that may influence effectiveness and programme engagement (including participant characteristics, setting, provider and programme delivery).^35 36^ Evaluating outcomes for up to 31 000 FNP families and up to 1 million controls will build on the results of the Building Blocks trial by providing increased statistical power to detect smaller differences, differences in rarer outcomes and subgroup differences for which the Building Blocks trial was underpowered. Study results will inform targeting of services and commissioning by generating evidence on which groups of mothers and their children benefit from the real-world implementation of FNP in England.

## Methods and analysis

### Study design and population

This study aims to evaluate the real-world, ongoing implementation of the FNP in England on the outcomes of mothers participating in FNP and their children, with the specific objectives of assessing (1) which groups of mothers receive FNP across LAs, (2) the effect of the FNP on maternal and child outcomes, including which families benefit most from FNP and (3) contextual factors influencing FNP programme effect. It will complement results from the Building Blocks trial in England,^27^ Building Blocks 2–6 year follow-up^31^ and evaluation of the FNP in Scotland using linked administrative data.^37^

We will create a retrospective cohort of all first-time mothers aged 13–24 years giving birth in England between 1 April 2010 and 31 March 2017 and their children, using individual-level, linked, longitudinal data from routinely collected hospital, education and social care records. Mothers up to 24 years will be included because some FNP sites modified inclusion criteria to include young mothers up to this age. The cohort will be created through linkage of hospital records (Hospital Episode Statistics (HES)), education and social care records (National Pupil Database (NPD)) and FNP programme data for mothers and their children. Our approach builds on previous linkage of education and health records and validated methods of linking hospital records for mothers and babies.^38 39^ The infrastructure and methodology used to create the linked database will be highly relevant for ongoing evaluation and monitoring of the FNP and other early interventions.

### Description of data sources

#### Hospital records: Hospital Episode Statistics (HES)

Hospital records for mothers aged 13–24 years and their children in England will be extracted from records of births and deliveries in HES. HES is a data warehouse containing details of all hospital admissions (from 1997), outpatient appointments (from 2003) and A&E visits (from 2010) at NHS hospitals in England.^40^ HES data have been used extensively in research. In addition to the birth record, we will link information from hospital admissions and A&E visits for mother and child (including up to 11 years before delivery for the mother, see online supplementary appendix). Information captured in HES includes administrative data (including admission dates, NHS trust, GP code), demographic information (including age, sex, ethnicity) and clinical information (diagnoses and procedures). A unique ‘HESID’ is assigned to enable episodes of care for the same individual to be combined. Diagnoses are coded by professional coders in hospitals using ICD-10 codes (International Classification of Disease, V.10); procedures are coded using OPCS-4 codes (Office of Population, Censuses and Surveys Classification of Surgical operations and procedures, V.4).^41 42^ Date and cause of death are obtained via linkage between HES and civil registration (deaths) data performed by NHS Digital. Based on previous methodological work, we will link delivery records for mothers and birth records for their children within HES to create a mother-baby HES cohort.^38^

10.1136/bmjopen-2020-038530.supp1 Supplementary data

#### Education and social care records: National Pupil Database (NPD)

The HES cohort of mother-baby pairs will be linked to education and social care records from the Department for Education for both mothers and children in the FNP and control groups (including information before delivery for mothers). Information on assessments, attainment and progression at each key stage is available for all pupils in state schools in England, alongside eligibility for free school meals, information about special educational needs and information about absences and exclusions. NPD data, including social care data, have been used extensively in research.^43^

For both mothers and their children in the cohort, we will link HES data to the following NPD databases:

- The yearly School Census (formerly PLASC), the Pupil Referral Unit (PRU) Census and Alternative Provision—including pupil-level information from 2002 for pupils aged 2–19+ on special educational needs, free school meal eligibility and information about absences and exclusions.
- The Children in Need Census (CINC)—including information on referrals to children’s social services, assessments carried out on these children and whether the children became the subject of a child protection plan, from 2008.^44^
- The Children Looked After (CLA) return—including information on all looked after children and recent care leavers in England, from 2005.^45 46^

For mothers only, we will link to Key Stages 2, 4 and 5 data, which includes teacher assessments and/or test results in Years 3–6 (ages 7–11), Year 11 (age 16) and Year 12/13 (ages 17–18). For children only, we will link to the Early Years Census and Early Years Foundation Stage Profile (EYFSP), including teacher assessments for 3 and 4 year olds (from 2008) and 2 year olds (from 2014), as well as to Key Stage 1 assessment data (ages 5–6).

A Unique Pupil Number (UPN) is used for linkage of CLA and CINC with the NPD. The UPN is usually assigned at first entry to a maintained school or nursery, typically around the age of four or five. Therefore, it is not possible to link the NPD to CLA data for children who are looked after only before they enter nursery/school or adopted children (who are provided with a new UPN).^47^ However, information on out-of-home care available for FNP participants at all ages will enable us to examine the scale of underestimation of out-of-home care before school in the administrative data.

#### Programme delivery: FNP Information System (FNP IS)

The HES cohort will be linked to the FNP IS to obtain information on participation in FNP. The FNP IS is a bespoke system that supports the implementation of the FNP programme in England, provided by NHS Digital under contract to the FNP National Unit on behalf of Public Health England. Data are reported in real time and are used locally by FNP teams and nationally by the FNP National Unit to monitor programme delivery and support quality improvement.

Data collected in the FNP IS include information from the mother and child collected at enrolment (by 28 weeks gestation at the latest, including mother’s age, marital status, living arrangements, education, employment, social care), 36 weeks gestation (including maternal health, alcohol, drugs and smoking), birth (including birth weight and gestational age) and at regular intervals until 24 months after birth (including child health and development, social care and other maternal baseline variables). Information on each visit is also collected (including date, length of visit, family nurse seen and referrals to other services). The FNP IS became functional in 2009, and data quality is reported to be high from 2010 onwards. FNP data have been used in previous research.^48 49^

FNP IS contains maternal and child identifiers at enrolment/birth: name, sex, date of birth, postcode, GP code and NHS number. When mothers graduate from the FNP, pseudonymised data are retained by the FNP National Unit only and identifiers are held solely on secure servers at NHS Digital. Since some identifiers might have changed since enrolment (eg, mother’s name, postcode), identifiers will first be updated using the Personal Demographic Service (PDS) within NHS Digital, so that the most relevant set of identifiers can be used for linkage.^50^

The time span covered by each data source (including look-back, study and follow-up periods) is described in the online supplementary appendix.

### Data linkage

**Figure 1:**
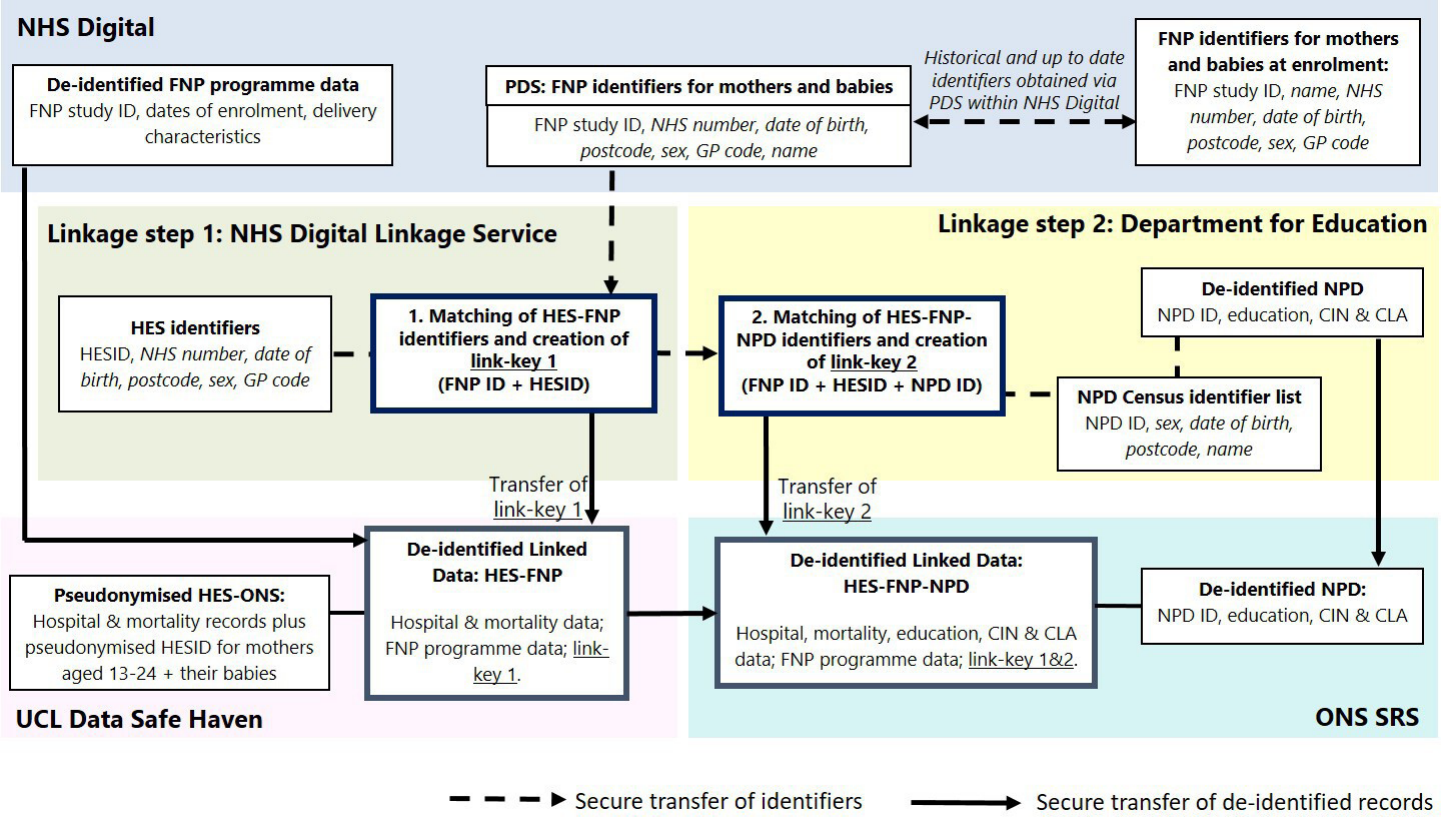
Data flows diagram. Note: items in italics are identifiers. CIN, Child in Need; CLA, Child Looked After; FNP, Family Nurse Partnership; GP, general practitioner; HES, Hospital Episode Statistics; NHS, National Health Service; NPD, National Pupil Database; ONS SRS, Office for National Statistics Secure Research Service; PDS, Personal Demographics Service; UCL, University College London.

Data flows are described in figure 1. Linkage of HES and FNP will be conducted by NHS Digital; linkage with NPD will be performed by the Department for Education. The linkage algorithm for each step (HES-FNP and HES-FNP-NPD) will be developed in an iterative process, taking into account the completeness of available identifiers (NHS number, GP practice, date of birth, sex, plus name for FNP participants) and building on existing strategies for linkage between HES and NPD. The quality of the linkage will be assessed by estimating the rates and distributions of linkage errors^39 51^ according to key maternal and child characteristics. Only deidentified data will be accessed by the research team, and the linked HES-FNP-NPD data will be securely stored on the Office for National Statistics Secure Research Service.

### Outcomes of interest

**Figure 2:**
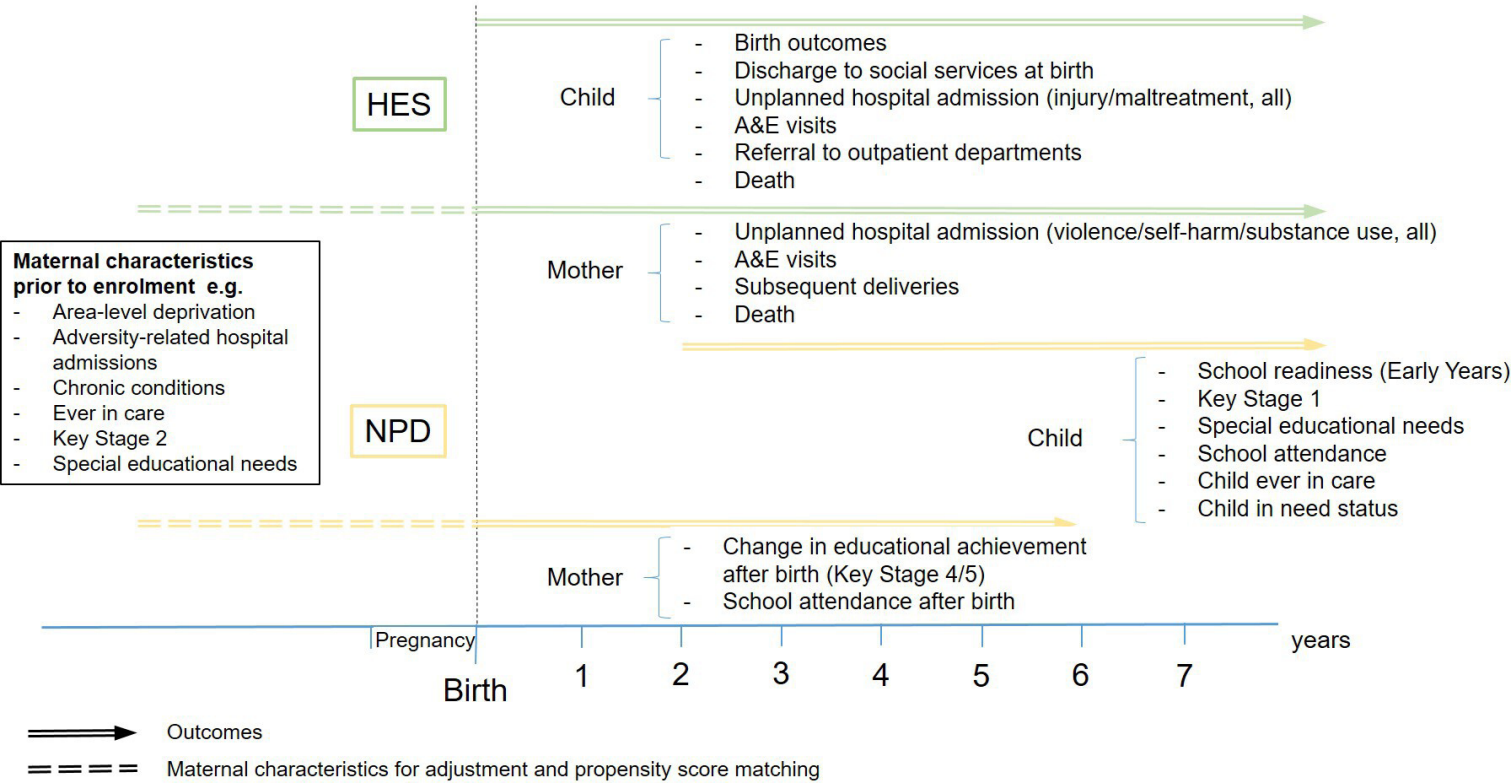
Family Nurse Partnership evaluation outcomes, data sources and selected maternal characteristics. HES, Hospital Episode Statistics; NPD, National Pupil Database.

Study outcomes are described in figure 2 and table 1. We selected outcomes for the FNP evaluation based on the FNP logic model,^52^ with some caveats outlined below. We will assess the FNP’s effect on child abuse and neglect, as measured by unplanned hospital admissions for injury or maltreatment-related diagnoses, child in need status, child looked after status and death. As the UPN for linking education and social care data is usually assigned at school entry, social care data for children only involved with social care prior to school entry cannot be linked. Therefore, we will only examine child in need and child looked after status after school starting age (4/5). We will also examine the FNP’s effect on other child outcomes including uptake and non-attendance for referral to outpatient departments, early years level of development, Key Stage 1 achievement, school attendance and Special Educational Needs status.

**Table 1:** Family Nurse Partnership evaluation outcomes and data sources

Domains	Outcomes	Years after birth	HES	NPD*	ONS
*Child outcomes (up to age 7)*					
Child abuse and neglect	Unplanned hospital admissions for any injury or maltreatment-related diagnosis (using ICD10 code lists)^66 67^ Discharge to social services at birth Child in care Child in need status Death	0–7 0 4/5–7 4/5–7 0–7	✓ ✓	✓ ✓	✓
Healthcare use	Unplanned hospital admissions (any diagnoses) A&E visits (any diagnoses) Referral to outpatient departments (uptake and non-attendance)	0–7 0–7 0–7	✓ ✓ ✓		
Education	Good level of development in early years assessment^68^ Key Stage 1 assessment SEN status School attendance	5 7 5–7 5–7		✓ ✓ ✓ ✓	
*Maternal outcomes*					
Maternal adversity	A&E visits (any diagnoses) Unplanned hospital admissions (any diagnoses, and for violence, self-harm or drug/alcohol abuse)^69^ Death	0–7 0–7 0–7	✓ ✓		✓
Reproductive outcomes	Subsequent deliveries within 18 months of index birth	0–2	✓		
Education	Key Stage 4 and 5 assessment† School attendance after birth‡	0–7 0–7		✓ ✓	

*Including the School Census, Child in Need Census and Child Looked After databases.

†Among mothers without Key Stage 4/5 attainment at birth.

‡School attendance information is collected up to age 15.

HES, Hospital Episode Statistics; NPD, National Pupil Database; ONS, Office for National Statistics; SEN, special educational needs.

Some healthcare use outcomes—A&E visits and unplanned hospital admissions for any diagnosis—will be reported as descriptive outcomes, since the direction of effect could be interpreted positively or negatively (FNP participation might reduce the need for emergency care or alternatively increase appropriate care-seeking). Nonetheless, they represent important outcomes for understanding the effects of FNP on care-seeking behaviour. Similarly, the potential for surveillance bias to distort the effect of early life interventions on child maltreatment has been discussed extensively:^19 27 53^ indeed, nurse home visiting results in increased contact with nurses, potentially leading to lower thresholds for reporting among participating families than control group families. This bias in ascertainment of maltreatment may dilute or reverse the association between FNP participation and maltreatment. Conversely, it has also been hypothesised that nurse’s closeness to participants may delay reporting of suspected maltreatment.^19^

Maternal outcomes will include unplanned hospital admissions for adversity-related reasons (violence, self-harm and drug/alcohol abuse) after birth and death. As for children, maternal rates of A&E visits and unplanned hospitalisations will be reported as descriptive outcomes. We will also examine the effect of FNP participation on school attendance and achievement after birth, and subsequent pregnancies within 18 months of the first birth ending in live or stillbirths. We will examine subsequent births from a pregnancy within 18 months (rather than 24 months as measured in the Building Blocks trial) because 18-month birth-to-pregnancy intervals are associated with the highest risks of adverse outcomes for women and babies.^54^ However, in addition to the outcomes in table 1, we will validate our findings against the outcomes reported in the Building Blocks trial by examining a range of birth outcomes and subsequent deliveries from pregnancies within 24 months of the index birth.

### Sample size and power calculation

Exploratory analysis of HES data suggests that approximately 9.5% of children born to first-time teenage mothers are admitted to hospital for injuries by age 6. Assuming a baseline rate of 9.5% for the percentage of children with this outcome up to age 6, a total of 6798 children (3399 in each group) would be required to observe a significant relative reduction of 20% in hospital admissions for injuries, with 80% power and a 5% significance level. In our cohort, birth outcomes will be available for around 31 000 FNP births; 24 000 FNP families will have a full 3 years of follow-up and 9000 will have a full 6 years of follow-up. The number of control (never enrolled) families will vary for the propensity score and adjusted analyses; up to one million potential controls (corresponding to total births to women aged 13–24 between April 2010 and March 2017) will be available for these analyses.

Key Stage 4 and 5 assessment outcomes (for ages 14–16 and 16–18, respectively) will be examined among mothers who have not sat these examinations before giving birth. We estimate that the sample size for this subgroup will be around 1800 FNP mothers for Key Stage 4 and 13 000 for Key Stage 5.

### Analysis

We will describe maternal sociodemographic characteristics at the time of pregnancy, previous health and educational characteristics and pregnancy outcomes for our retrospective cohort of teenage and young mothers (described above).

To determine whether the teenage mothers most in need receive FNP (objective 1), we will identify the proportion of eligible families participating in FNP by LA and over time. Predictors of participation will be derived from HES and NPD data. Since recruiting practices vary locally, we will compare data from high-coverage and low-coverage LAs, to explore whether FNP families in low-coverage LAs are more highly selected than those within LAs offering the programme to more mothers (eg, it may be hypothesised that nurses in some areas may target the most advantaged mothers or use a first-come-first-served model in others).

To examine the effects of the FNP (objective 2), we will compare outcomes for mothers ever enrolled in FNP, and their children, versus those never enrolled, using two analysis strategies to account for measured confounders related to FNP participation and outcomes. Propensity score matching aims to minimise bias, while adjustment for confounders aims to minimise variance.

#### Propensity score matching

To derive propensity scores, we will construct logistic regression models with FNP participation as the outcome, including as predictors all available maternal characteristics up to 28 weeks gestation (at which point the vast majority of mothers have been enrolled), including looked after status, educational achievement and pre-pregnancy chronic conditions. Matched groups will be formed by matching FNP participants to eligible non-participants with a similar participation propensity score.^55^ Effects will be estimated as the difference in outcomes between matched groups. Multiple imputation will be used to account for missing data where data are expected to be missing at random.^56^

The main analysis will restrict matching within the same LA^57^ and time period in which FNP was offered within that LA. Secondary analyses aiming to achieve more closely matched groups (with potentially smaller numbers) will match, first, within the same LA but in different time periods, comparing outcomes for eligible families before versus after FNP was offered; and second, within the same time period but in different LAs, comparing outcomes for eligible families in LAs that did and did not offer FNP.

#### Adjusted analyses

This analysis will be an unmatched comparison, adjusting for maternal characteristics prior to enrolment for each outcome based on causal diagrams and hypothesised influence on effect estimates.

Sensitivity analyses will determine the strength of unmeasured confounding required to invalidate results.^58 59^ To further assess the robustness of findings to the analysis approach and to evaluate any potential differences in results due to the use of real-world data, we will use our cohort to replicate findings observed in the Building Blocks trial. For each analysis strategy, we will derive trial outcomes for a group of families in the administrative data cohort with the same aggregate baseline characteristics as trial participants.

Stratified analyses and interaction terms will be used to investigate effect modification according to the maternal characteristics in box 1, based on previous evidence suggesting the youngest and most disadvantaged mothers are most likely to benefit from the FNP.

Box 1 **Maternal characteristics prior to enrolment (Family Nurse Partnership participants) or up to 28 weeks gestation (controls) potentially producing effect modification**

- Maternal age at enrolment.
- Ethnicity.
- Healthcare use prior to enrolment (unplanned admissions and A&E visits, admissions for drug/alcohol abuse, violence or self-harm).^69^
- Chronic conditions (including mental health conditions captured in hospital data).^70^
- Educational achievement.
- Special educational needs.
- Eligibility for free school meals.
- Out-of-home care.
- Area-level deprivation.

To identify contextual factors associated with benefitting from FNP (objective 3), we will explore individual and programme characteristics associated with programme engagement, including maternal characteristics prior to enrolment, nurse characteristics and usual care models in LAs. We will also investigate any differences in FNP effect between LAs and between our cohort and the corresponding Building Blocks trial population (restricted to trial inclusion criteria), to help generalise trial results.

A detailed statistical plan will be written prior to analyses. Statistical models will take into account clustering within LAs, and also among nurses where appropriate. Data will be analysed in Stata. Study results will be reported in accordance with GUILD, STROBE and RECORD guidelines.^60–62^

### Limitations

We do not have individual-level data on primary care or the number of visits from health visitors that families receive outside of FNP; however, we will seek local aggregate data on usual care models of health visiting and targeted support for teenage mothers over time to examine how these differed across LAs.

Data on mothers who were offered FNP but declined are not routinely collected. Women who refuse participation may therefore be included in our control group; this may introduce some selection bias, since refusers are likely to differ from FNP accepters in ways that also affect their outcomes. However, propensity score matching sensitivity analyses will restrict controls to women who could not have been offered FNP (either before FNP was offered or in LAs not offering FNP). In the event of remaining unmeasured confounders, grouping refusers with other non-participants may result in an underestimation of FNP effects (if less vulnerable women are more likely to refuse) or overestimation (if more vulnerable women are more likely to refuse). Although individual-level data on refusers are not available, we will obtain data from FNP sites, where available, on the rate and aggregate characteristics of mothers declining participation.

FNP eligibility criteria require enrolment before 28 weeks gestation. Due to a high proportion of missing data on gestational age at first antenatal booking appointment, we will include women who are missing this information as potential controls. Ninety-two per cent of all women present by 20 weeks gestation;^63^ therefore, any resulting bias in results is likely to be small. However, we will also conduct sensitivity analyses restricting the control group to women presenting up to 28 weeks at booking appointment, to match FNP eligibility criteria.

We estimate at least 90% completeness for linkage of HES and NPD. There may be selection bias in the analyses related to education and social care outcomes due to mothers or children who are not linked being different to those whose records are successfully linked.^51^ Nonetheless, data completeness in our cohort is likely to be substantially higher than follow-up rates for medium-term outcomes in most existing trials^15 64 65^ (although the Building Blocks trial in England is also using linkage of routine records for follow-up to the child’s seventh birthday).^31^ Linkage of mother-baby records within HES is high (98%).^38^

## Ethics and dissemination

### Patient and public involvement

In the process of designing our study, we engaged with two groups of mothers (some who had participated in FNP, some who had not). We discussed the use of administrative data for research (including concepts that mothers were not previously familiar with, such as deidentification and data linkage), linkage of health and education data without explicit consent and the use of these data specifically for evaluating the FNP. Workshop participants were supportive of the proposed study and fed back that linking health and education data for mothers and their children was a good idea. Participants strongly agreed with sharing their data so that services could be improved and future mothers could benefit, and wanted to know how their data had been used to benefit others. Mothers were strongly supportive of taking into account maternal education and area, and wider family support for the FNP, in order to understand whether the programme worked. We incorporated these opinions into our study plan by planning to evaluate whether the effect of FNP differs by area and by maternal characteristic.

Our Study Steering Committee includes a teenage mother, who will continue to be consulted throughout the study period. Parent participants will help coproduce information on the study for the FNP and institutional websites and help with interpretation of results and identifying the most appropriate methods of dissemination.

### Dissemination

This study will generate policy-relevant findings on the implementation of the FNP in England and likely benefits to maternal and child health, building on evidence from the Building Blocks trial of FNP. By evaluating outcomes for the 31 000 families enrolled in FNP between 2010 and 2017, our study will determine whether there is evidence of benefits for additional outcomes up to age 7 and within subgroups (including especially vulnerable young mothers and children). Our findings will directly benefit the Health and Social Care sector by providing LAs, commissioners and policy-makers with detailed and up-to-date evidence to inform the ongoing rollout and targeting of early interventions designed to support young mothers and child development.

Our main output will consist of evidence on the effectiveness of FNP for different groups of families. These results will be disseminated to policy-makers and commissioners in collaboration with the FNP National Unit and will inform ongoing research into the adaptation of the FNP in England. We will work with parent representatives to coproduce a range of outputs suitable for families participating in FNP. For academic beneficiaries and other researchers, we aim to publish our main findings in high-quality, peer-reviewed journals as well as present at key conferences. Secondary outputs will include methodological research on the accuracy and reliability of data linkage from health, education and social care sectors, which will inform the use of administrative data by data providers and other researchers.

## Supplementary Material

Reviewer comments

Author's manuscript
