# Supplementary material for: Evaluating the real-world implementation of the Family Nurse Partnership in England: protocol for a data linkage study
Source: BMJ Open. 2020 May 18;10(5):e038530. doi: 10.1136/bmjopen-2020-038530 (PMC7239518; doi:10.1136/bmjopen-2020-038530)
Supplement: Supplementary data [file bmjopen-2020-038530supp001.pdf]

Appendix

Time periods covered by data sources. Shaded boxes indicate cohort member age during study period (blue); look-back period (grey); follow-up (light blue).

	Data source	Year of data																		
		2001	2002	2003	2004	2005	2006	2007	2008	2009	2010	2011	2012	2013	2014	2015	2016	2017	2018	2019
	Births																			
	FNP																			
	HES																			
	NPD																			
	CLA																			
	CIN																			
Year of birth of cohort member	1986	15	16	17	18	19	20	21	22	23	24	25	26	27	28	29	30	31	32	33
	1987	14	15	16	17	18	19	20	21	22	23	24	25	26	27	28	29	30	31	32
	1988	13	14	15	16	17	18	19	20	21	22	23	24	25	26	27	28	29	30	31
	1989	12	13	14	15	16	17	18	19	20	21	22	23	24	25	26	27	28	29	30
	1990	11	12	13	14	15	16	17	18	19	20	21	22	23	24	25	26	27	28	29
	1991	10	11	12	13	14	15	16	17	18	19	20	21	22	23	24	25	26	27	28
	1992	9	10	11	12	13	14	15	16	17	18	19	20	21	22	23	24	25	26	27
	1993	8	9	10	11	12	13	14	15	16	17	18	19	20	21	22	23	24	25	26
	1994	7	8	9	10	11	12	13	14	15	16	17	18	19	20	21	22	23	24	25
	1995	6	7	8	9	10	11	12	13	14	15	16	17	18	19	20	21	22	23	24
	1996	5	6	7	8	9	10	11	12	13	14	15	16	17	18	19	20	21	22	23
	1997	4	5	6	7	8	9	10	11	12	13	14	15	16	17	18	19	20	21	22
	1998	3	4	5	6	7	8	9	10	11	12	13	14	15	16	17	18	19	20	21
	1999	2	3	4	5	6	7	8	9	10	11	12	13	14	15	16	17	18	19	20
	2000	1	2	3	4	5	6	7	8	9	10	11	12	13	14	15	16	17	18	19
	2001	0	1	2	3	4	5	6	7	8	9	10	11	12	13	14	15	16	17	18
	2002		0	1	2	3	4	5	6	7	8	9	10	11	12	13	14	15	16	17
	2003			0	1	2	3	4	5	6	7	8	9	10	11	12	13	14	15	16
	2004				0	1	2	3	4	5	6	7	8	9	10	11	12	13	14	15
	2005					0	1	2	3	4	5	6	7	8	9	10	11	12	13	14
	2006						0	1	2	3	4	5	6	7	8	9	10	11	12	13
	2007							0	1	2	3	4	5	6	7	8	9	10	11	12
	2008								0	1	2	3	4	5	6	7	8	9	10	11
	2009									0	1	2	3	4	5	6	7	8	9	10
	2010										0	1	2	3	4	5	6	7	8	9
	2011											0	1	2	3	4	5	6	7	8
	2012												0	1	2	3	4	5	6	7
	2013													0	1	2	3	4	5	6
	2014														0	1	2	3	4	5
	2015															0	1	2	3	4
	2016																0	1	2	3
	2017																	0	1	2
