# Supplementary material for: Evaluating the real-world implementation of the Family Nurse Partnership in England: protocol for a data linkage study
Source: BMJ Open. 2020 May 18;10(5):e038530. doi: 10.1136/bmjopen-2020-038530 (PMC7239518; doi:10.1136/bmjopen-2020-038530)
Supplement: Author's manuscript [file bmjopen-2020-038530.draft_revisions.pdf]

BMJ Open is committed to open peer review. As part of this commitment we make the peer review history of every article we publish publicly available.

When an article is published we post the peer reviewers' comments and the authors' responses online. We also post the versions of the paper that were used during peer review. These are the versions that the peer review comments apply to.

The versions of the paper that follow are the versions that were submitted during the peer review process. They are not the versions of record or the final published versions. They should not be cited or distributed as the published version of this manuscript.

BMJ Open is an open access journal and the full, final, typeset and author-corrected version of record of the manuscript is available on our site with no access controls, subscription charges or pay-per-view fees (http://bmjopen.bmj.com).

If you have any questions on BMJ Open's open peer review process please email info.bmjopen@bmj.com

BMJ Open

Evaluating the real-world implementation of the Family Nurse Partnership in England: protocol for a data linkage study

Journal:	*BMJ Open*
Manuscript ID	bmjopen-2020-038530
Article Type:	Protocol
Date Submitted by the Author:	13-Mar-2020
Complete List of Authors:	Cavallaro, Francesca; University College London, Institute of Child Health<br>Gilbert, Ruth; University College London, Institute of Child Health<br>Wijlaars, Linda; University College London, Institute of Child Health<br>Kennedy, Eilis; Tavistock and Portman NHS Foundation Trust<br>Swarbrick, Ailsa; Tavistock and Portman NHS Foundation Trust<br>van der Meulen, Jan; London School of Hygiene & Tropical Medicine, Department of Health Services Research and Policy<br>Harron, Katie; University College London, Institute of Child Health
Keywords:	Community child health < PAEDIATRICS, Child protection < PAEDIATRICS, PUBLIC HEALTH, Health informatics < BIOTECHNOLOGY & BIOINFORMATICS

SCHOLARONE™
Manuscripts

I, the Submitting Author has the right to grant and does grant on behalf of all authors of the Work (as defined in the below author licence), an exclusive licence and/or a non-exclusive licence for contributions from authors who are: i) UK Crown employees; ii) where BMJ has agreed a CC-BY licence shall apply, and/or iii) in accordance with the terms applicable for US Federal Government officers or employees acting as part of their official duties; on a worldwide, perpetual, irrevocable, royalty-free basis to BMJ Publishing Group Ltd ("BMJ") its licensees and where the relevant Journal is co-owned by BMJ to the co-owners of the Journal, to publish the Work in this journal and any other BMJ products and to exploit all rights, as set out in our licence.

The Submitting Author accepts and understands that any supply made under these terms is made by BMJ to the Submitting Author unless you are acting as an employee on behalf of your employer or a postgraduate student of an affiliated institution which is paying any applicable article publishing charge ("APC") for Open Access articles. Where the Submitting Author wishes to make the Work available on an Open Access basis (and intends to pay the relevant APC), the terms of reuse of such Open Access shall be governed by a Creative Commons licence – details of these licences and which Creative Commons licence will apply to this Work are set out in our licence referred to above.

Other than as permitted in any relevant BMJ Author's Self Archiving Policies, I confirm this Work has not been accepted for publication elsewhere, is not being considered for publication elsewhere and does not duplicate material already published. I confirm all authors consent to publication of this Work and authorise the granting of this licence.

For peer review only

Evaluating the real-world implementation of the Family Nurse Partnership in England: protocol for a data linkage study

Authors: Francesca L Cavallaro,[1] Ruth Gilbert,[1] Linda Wijlaars,[1] Eilis Kennedy,[2] Ailsa Swarbrick,[2] Jan van der Meulen,[3] Katie L Harron[1]*

[1]UCL GOS Institute of Child Health, London, UK

[2]Tavistock and Portman NHS Foundation Trust, London, UK

[3]Department of Health Services Research and Policy, London School of Hygiene & Tropical Medicine, London, UK

*correspondence: Dr Katie Harron, k.harron@ucl.ac.uk

For peer review only

Abstract

Introduction. Almost 20,000 babies are born to teenage mothers each year in England, with poorer outcomes for mothers and babies than among older mothers. A nurse home visitation programme in the US was found to improve a wide range of outcomes for young mothers and their children. However, a randomised controlled trial in England found no effect on short-term primary outcomes, although cognitive development up to age two showed improvement. Our study will use linked routinely collected health, education and social care data to evaluate the real-world effects of the Family Nurse Partnership (FNP) on child outcomes up to age seven, with a focus on identifying whether the FNP works better for particular groups of families, thereby informing programme targeting and resource allocation.

Methods and analysis. We will construct a retrospective cohort of all women aged 13-24 years giving birth in English NHS hospitals between 2010 and 2017, linking information on mothers and children from FNP programme data, Hospital Episodes Statistics and the National Pupil Database. To assess the effectiveness of FNP, we will compare outcomes for eligible mothers ever and never enrolled in FNP, and their children, using two analysis strategies to adjust for measured confounding: propensity score matching and analyses adjusting for maternal characteristics up to enrolment / 28 weeks gestation. Outcomes of interest include early childhood development, childhood unplanned hospital admissions for injury or maltreatment-related diagnoses, and children in care. Subgroup analyses will determine whether the effect of FNP varied according to maternal characteristics (e.g. age and education).

Ethics and dissemination. The Nottingham Research Ethics Committee approved this study. Mothers participating in FNP were supportive of our planned research. Results will inform policy-makers for targeting home visiting programmes. Methodological findings on the accuracy and reliability of cross-sectoral data linkage will be of interest to researchers.

Keywords: Health informatics, community child health, child protection, public health

Word count: 4,296

Strengths and limitations of this study

- This study will complement results from the Building Blocks trial in England, by evaluating the real-world effect of the Family Nurse Partnership (FNP) on a range of health, educational and social care outcomes up to age 7 years.
- The study cohort comprises joint trajectories for mothers and babies captured in Hospital Episode Statistics (HES; containing >97% births in England and information on subsequent healthcare use), with linked information to FNP programme data and the National Pupil Database (NPD; containing information on children's and mothers' educational attainment and social care).
- Evaluating outcomes for up to 31,000 FNP families and up to 1 million controls will provide statistical power to detect small differences, differences in rare outcomes, and to perform subgroup analysis to determine whether the FNP is more effective in particular groups of families.
- No unique identifiers exist between HES and NPD databases, and linkage may be incomplete.
- Findings will inform the adaptation of the FNP and targeting of support for teenage and young mothers to specific subgroups.

For peer review only

For peer review only - http://bmjopen.bmj.com/site/about/guidelines.xhtml

Introduction

Approximately 3% of babies each year (19,000 in 2018) are born to mothers aged below 20 in England and Wales.[1] Teenage mothers are more likely to experience adversity, be less engaged with education and employment, and have rapid repeat pregnancies compared with older mothers.[2-5] For their children, young maternal age is associated with higher incidence of preterm birth and low birthweight,[6 7] and a greater risk of child maltreatment and associated adverse long-term consequences including poorer physical, social, emotional and cognitive outcomes.[8-10] These adverse maternal and child outcomes of teenage pregnancy, due to social adversity, disruption to education and employment, and child-rearing practices, are of major importance to public health research.[11 12]

The Family Nurse Partnership (FNP) is an intensive home visiting programme that aims to improve outcomes of pregnancies among vulnerable young mothers. Participants receive up to 64 home visits from early pregnancy until the child's second birthday. Specifically, the FNP aims to improve birth outcomes, optimise child health and development, and promote economic self-sufficiency among vulnerable mothers.[13] Three randomised controlled trials in the US found evidence of benefit on a number of maternal and child outcomes, including reductions in child maltreatment, child emergency department visits, and subsequent births, as well as some improvements in child developmental outcomes and longer subsequent birth intervals.[14-18] Effects on hospital visits for injury or maltreatment, children's educational achievements and maternal educational qualifications were less consistent. A trial in the Netherlands found reduced child maltreatment reports by age 2,[19] and a trial of a FNP-based model in Germany reported improved child development among high-risk women only, although no difference in subsequent births within two years.[20] Non-randomised studies have reported reduced child maltreatment,[21] infants death,[22] and subsequent births[23] among FNP participants compared to controls, as well as different patterns of emergency room visits.[24]

Following evidence from the US trials, FNP was introduced in the UK in 2007. The FNP was rolled out via partnerships between local authorities (LA) and healthcare organisations; to date, more than 130 local authorities have implemented the programme. Teenage and young mothers who do not participate in the FNP receive usual care, consisting in comprehensive maternity care and at least five home visits by a health visitor at a minimum;[25] substantial variation in usual care exists between LAs.[26] In England, the FNP was initially offered to pregnant teenagers aged under 20 years up to 28 weeks gestation, but since 2017 selected sites have expanded to include women up to 24 years with specific vulnerability criteria, and/or have implemented some personalisation of the programme.

Following trials in other countries, the Building Blocks trial randomised 1,618 families in England between 2009 and 2010 and showed no benefit of FNP on primary outcomes up to the child's second birthday

For peer review only - http://bmjopen.bmj.com/site/about/guidelines.xhtml

(smoking in late pregnancy, birthweight, subsequent pregnancy, or child A&E and hospital attendances).[27] However, the interpretation of trial results was debated due to the choice of maternally-reported early childhood development as a secondary outcome, for which a positive effect was observed, and strong support locally for the value of FNP on parent-child relationships.[28-30] Follow-up has been extended up to the child's seventh birthday, with a focus on child maltreatment.[31]

Evaluating complex interventions such as the FNP requires an understanding of the context in which they are delivered as well as the mechanisms of change, in order to investigate and interpret any differences in effects across contexts and populations.[32 33] Indeed, differences in the comparator of usual care may explain differences in results between trials in the US, England, and the Netherlands; these may also vary over time and between LAs in England.[26 28] Furthermore, evidence from the US trials suggests that the youngest, most disadvantaged mothers are likely to benefit most from FNP.[28] Adequately powered subgroup analyses are needed to examine whether some groups of families benefited from FNP more than others. In addition, constrained conditions under which trials are conducted often do not match the complexity of real-world implementation of programmes.[34]

Linkage of existing administrative records provides a cost-efficient means of evaluating services as they are implemented in the real world, by bringing together data from different sectors on a range of outcomes. They also allow for a sufficiently large sample size for subgroup analyses. Our population-based study will use longitudinal observational data to evaluate effects on outcomes of mothers and children up to age seven among eligible families, and generate evidence on the factors that may influence effectiveness and programme engagement (including participant characteristics, setting, provider, and programme delivery).[35 36] Evaluating outcomes for up to 31,000 FNP families and up to 1 million controls will build on the results of the Building Blocks trial by providing increased statistical power to detect smaller differences, differences in rarer outcomes, and subgroup differences for which the Building Blocks trial was underpowered. Study results will inform targeting of services and commissioning by generating evidence on which groups of mothers and their children benefit from the real-world implementation of FNP in England.

Methods and analysis

Study design and population

This study aims to evaluate the real-world, ongoing implementation of the FNP in England on the outcomes of mothers participating in FNP and their children, with the specific objectives of assessing (1) which groups of mothers receive FNP across LAs, (2) the effect of the FNP on maternal and child outcomes, including which families benefit most from FNP, and (3) contextual factors influencing FNP programme effect. It will

For peer review only

complement results from the Building Blocks trial in England,[27] Building Blocks 2-6 year follow-up,[31] and evaluation of the FNP in Scotland using linked administrative data.[37]

We will create a retrospective cohort of all first-time mothers aged 13-24 years delivering a live birth in England between 1st April 2010 and 31st March 2017 and their children, using individual-level, linked, longitudinal data from routinely collected hospital, education and social care records. Mothers up to 24 years will be included because some FNP sites modified inclusion criteria to include young mothers up to this age. The cohort will be created through linkage of hospital records (Hospital Episode Statistics; HES), education and social care records (National Pupil Database; NPD), and FNP programme data for mothers and their children. Our approach builds on previous linkage of education and health records, and validated methods of linking hospital records for mothers and babies.[38 39] The infrastructure and methodology used to create the linked database will be highly relevant for ongoing evaluation and monitoring of the FNP and other early interventions.

Description of data sources

Hospital records: Hospital Episode Statistics (HES)

Hospital records for mothers aged 13-24 years and their children in England will be extracted from records of births and deliveries in HES. HES is a data warehouse containing details of all hospital admissions (from 1997), outpatient appointments (from 2003) and A&E visits (from 2010) at NHS hospitals in England.[40] HES data have been used extensively in research. In addition to the birth record, we will link information from hospital admissions and A&E visits for mother and child (including up to 11 years before delivery for the mother, see Appendix). Information captured in HES includes administrative data (including admission dates, NHS trust, GP code), demographic information (including age, sex, ethnicity), and clinical information (diagnoses and procedures). A unique 'HESID' is assigned to enable episodes of care for the same individual to be combined. Diagnoses are coded by professional coders in hospitals using ICD-10 codes (International Classification of Disease, version 10); procedures are coded using OPCS-4 codes (Office of Population, Censuses and Surveys Classification of Surgical operations and procedures, version 4).[41 42] Date and cause of death are obtained via linkage between HES and civil registration (deaths) data performed by NHS Digital. Based on previous methodological work, we will link delivery records for mothers and birth records for their children within HES to create a mother-baby HES cohort.[38]

Education and social care records: National Pupil Database (NPD)

The HES cohort of mother-baby pairs will be linked to education and social care records from the Department for Education for both mothers and children in the FNP and control groups (including information before delivery for mothers). Information on assessments, attainment and progression at each

For peer review only - http://bmjopen.bmj.com/site/about/guidelines.xhtml

key stage is available for all pupils in state schools in England, alongside eligibility for free school meals, information about special educational needs and information about absences and exclusions. NPD data, including social care data, have been used extensively in research.[43]

For both mothers and their children in the cohort, we will link HES data to the following NPD databases:

- The yearly School Census (formerly PLASC), the Pupil Referral Unit (PRU) Census, and Alternative Provision – including pupil-level information from 2002 for pupils aged 2-19+ on special educational needs, free school meal eligibility and information about absences and exclusions.
- The Children in Need Census (CINC) – including information on referrals to children's social services, assessments carried out upon these children, and whether the children became the subject of a child protection plan, from 2008.[44]
- The Children Looked After (CLA) return – including information on all looked after children and recent care leavers in England, from 2005.[45 46]

For mothers only, we will link to Key Stages 2, 4 and 5 data, which includes teacher assessments and/or test results in Years 3-6 (ages 7-11), Year 11 (age 16) and Year 12/13 (ages 17-18). For children only, we will link to the Early Years Census and Early Years Foundation Stage Profile (EYFSP), including teacher assessments for three and four year olds (from 2008), and two year olds (from 2014), as well as to Key Stage 1 assessment data (ages 5-6).

A Unique Pupil Number (UPN) is used for linkage of CLA and CINC with the NPD. The UPN is usually assigned at first entry to a maintained school or nursery, typically around the age of four or five. Therefore it is not possible to link the NPD to CLA data for children who are looked after only before they enter nursery/school, or adopted children (who are provided with a new UPN).[47] However, information on out-of-home care available for FNP participants at all ages will enable us to examine the scale of underestimation of out-of-home care before school in the administrative data.

Programme delivery: FNP Information System (FNP IS)

The HES cohort will be linked to the FNP IS to obtain information on participation in FNP. The FNP IS is a bespoke system that supports the implementation of the FNP programme in England, provided by NHS Digital under contract to the FNP National Unit on behalf of Public Health England. Data are reported in real time and are used locally by FNP teams and nationally by the FNP National Unit to monitor programme delivery and support quality improvement.

Data collected in the FNP IS include information from the mother and child collected at enrolment (by 28 weeks gestation at the latest, including mother's age, marital status, living arrangements, education, employment, social care), 36 weeks gestation (including maternal health, alcohol, drugs and smoking), birth (including birthweight and gestational age) and at regular intervals until 24 months after birth (including

For peer review only - http://bmjopen.bmj.com/site/about/guidelines.xhtml

child health and development, social care and other maternal baseline variables). Information on each visit is also collected (including date, length of visit, family nurse seen, and referrals to other services). The FNP IS became functional in 2009, and data quality is reported to be high from 2010 onwards. FNP data have been used in previous research.[48 49]

FNP IS contains maternal and child identifiers at enrolment/birth: name, sex, date of birth, postcode, GP code and NHS number. When mothers graduate from the FNP, pseudonymised data are retained by the FNP National Unit only and identifiers are held solely on secure servers at NHS Digital. Since some identifiers might have changed since enrolment (e.g. mother's name, postcode), identifiers will first be updated using the Personal Demographic Service (PDS) within NHS Digital, so that the most relevant set of identifiers can be used for linkage.[50]

The time span covered by each data source (including look-back, study and follow-up periods) is described in the Appendix.

Data linkage

Data flows are described in Figure 1. Linkage of HES and FNP will be conducted by NHS Digital; linkage with NPD will be performed by the Department for Education. The linkage algorithm for each step (HES-FNP, and HES-FNP-NPD) will be developed in an iterative process, taking into account the completeness of available identifiers (NHS number, GP practice, date of birth, sex, plus name for FNP participants) and building on existing strategies for linkage between HES and NPD. The quality of the linkage will be assessed by estimating the rates and distributions of linkage errors[39 51] according to key maternal and child characteristics. Only de-identified data will be accessed by the research team, and the linked HES-FNP-NPD data will be securely stored on the Office for National Statistics Secure Research Service (ONS SRS).

Outcomes of interest

Study outcomes are described in Figure 2 and Table 1. We selected outcomes for the FNP evaluation based on the FNP logic model,[52] with some caveats outlined below. We will assess the FNP's effect on child abuse and neglect, as measured by unplanned hospital admissions for injury or maltreatment-related diagnoses, child in need status, child looked after status, and death. As the UPN for linking education and social care data is usually assigned at school entry, social care data for children only involved with social care prior to school entry cannot be linked. Therefore, we will only examine child in need and child looked after status after school starting age (4/5). We will also examine the FNP's effect on other child outcomes including uptake and non-attendance for referral to outpatient departments, early years level of development, Key Stage 1 achievement, school attendance, and Special Educational Needs status.

For peer review only - http://bmjopen.bmj.com/site/about/guidelines.xhtml

Some healthcare use outcomes – A&E visits and unplanned hospital admissions for any diagnosis – will be reported as descriptive outcomes, since the direction of effect could be interpreted positively or negatively (FNP participation might reduce the need for emergency care, or alternatively increase appropriate care-seeking). Nonetheless, they represent important outcomes for understanding the effects of FNP on care-seeking behaviour. Similarly, the potential for surveillance bias to distort the effect of early life interventions on child maltreatment has been discussed extensively:[19 27 53] indeed, nurse home visiting results in increased contact with nurses, potentially leading to lower thresholds for reporting among participating families than control group families. This bias in ascertainment of maltreatment may dilute or reverse the association between FNP participation and maltreatment. Conversely, it has also been hypothesised that nurse's closeness to participants may delay reporting of suspected maltreatment.[19]

Maternal outcomes will include unplanned hospital admissions for adversity-related reasons (violence, self-harm, and drug/alcohol abuse) after birth, and death. As with child outcomes, rates of A&E visits and unplanned hospitalisations will be reported as descriptive outcomes. We will also examine the effect of FNP participation on school attendance and achievement after birth, and subsequent pregnancies within 18 months of the first birth ending in live or stillbirths. We will examine subsequent births from a pregnancy within 18 months (rather than 24 months as measured in the Building Blocks trial) because 18 month birth-to-pregnancy intervals are associated with the highest risks of adverse outcomes for women and babies.[54] However, in addition to the outcomes in Table 1, we will validate our findings against the outcomes reported in the Building Blocks trial by examining a range of birth outcomes and subsequent deliveries from pregnancies within 24 months of the index birth.

For peer review only - http://bmjopen.bmj.com/site/about/guidelines.xhtml

Table 1. Family Nurse Partnership evaluation outcomes and data sources

Domains	Outcomes	Years after birth	HES	NPD[a]	ONS
**Child outcomes (up to age 7)**					
Child abuse and neglect	Unplanned hospital admissions for any injury or maltreatment-related diagnosis (using ICD10 code lists)[55 56]	0-7	✓		
	Discharge to social services at birth	0	✓		
	Child in care	4/5-7		✓	
	Child in need status	4/5-7		✓	
	Death	0-7			✓
Healthcare use	Unplanned hospital admissions (any diagnoses)	0-7	✓		
	A&E visits (any diagnoses)	0-7	✓		
	Referral to outpatient departments (uptake and non-attendance)	0-7	✓		
Education	Good level of development in early years assessment[57]	5		✓	
	Key Stage 1 assessment	7		✓	
	Special educational needs (SEN) status	5-7		✓	
	School attendance	5-7		✓	
**Maternal outcomes**					
Maternal adversity	A&E visits (any diagnoses)	0-7	✓		
	Unplanned hospital admissions (any diagnoses, and for violence, self-harm, or drug/alcohol abuse)[58]	0-7	✓		
	Death	0-7			✓
Reproductive outcomes	Subsequent deliveries within 18 months of index birth	0-2	✓		
Education	Key Stage 4 and 5 assessment[b]	0-7		✓	
	School attendance after birth[c]	0-7		✓	

[a]Including the School Census, Child in Need Census and Child Looked After databases; [b]Among mothers without Key Stage 4/5 attainment at birth; [c]School attendance information is collected up to age 15

Sample size and power calculation

Exploratory analysis of HES data suggests that approximately 9.5% of children born to first time teenage mothers are admitted to hospital for injuries by age 6. Assuming a baseline rate of 9.5% for the percentage of children with this outcome up to age 6, a total of 6,798 children (3,399 in each group) would be required to observe a significant relative reduction of 20% in hospital admissions for injuries, with 80% power and a 5% significance level. In our cohort, birth outcomes will be available for around 31,000 FNP births; 24,000 FNP families will have a full 3 years of follow-up and 9,000 will have a full 6 years of follow up. The number of control (never enrolled) families will vary for the propensity score and adjusted analyses; up to one million potential controls (corresponding to total births to women aged up to 24 between April 2010 and March 2017) will be available for these analyses.

Key Stage 4 and 5 assessment outcomes (for ages 14-16 and 16-18, respectively) will be examined among mothers who have not sat these exams before giving birth. We estimate that the sample size for this subgroup will be around 1,800 FNP mothers for Key Stage 4 and 13,000 for Key Stage 5.

For peer review only - http://bmjopen.bmj.com/site/about/guidelines.xhtml

Analysis

We will describe maternal socio-demographic characteristics at the time of pregnancy, previous health and educational characteristics, and pregnancy outcomes for our retrospective cohort of teenage and young mothers (described above).

To determine whether the teenage mothers most in need receive FNP (objective 1), we will identify the proportion of eligible families participating in FNP by LA and over time. Predictors of participation will be derived from HES and NPD data. Since recruiting practices vary locally, we will compare data from high- and low-coverage LAs, to explore whether FNP families in low-coverage LAs are more highly selected than those within LAs offering the programme to more mothers (for example, it may be hypothesised that nurses in some areas may target the most advantaged mothers, or use a first-come-first-served model in others).

To examine the effects of the FNP (objective 2), we will compare outcomes for mothers ever enrolled in FNP, and their children, versus those never enrolled, using two analysis strategies to account for measured confounders related to FNP participation and outcomes. Propensity score matching aims to minimise bias, while adjustment for confounders aims to minimise variance.

1. Propensity score matching

To derive propensity scores, we will construct logistic regression models with FNP participation as the outcome, including as predictors all available maternal characteristics up to 28 weeks gestation (at which point the vast majority of mothers have been enrolled), including looked after status, educational achievement, and pre-pregnancy chronic conditions. Matched groups will be formed by matching FNP participants to eligible non-participants with a similar participation propensity score.[59] Effects will be estimated as the difference in outcomes between matched groups. Multiple imputation will be used to account for missing data where data are missing at random.[60]

The main analysis will restrict matching within the same LA[61] and time period in which FNP was offered within that LA. Secondary analyses aiming to achieve more closely matched groups (with potentially smaller numbers) will match, first, within the same LA but in different time periods, comparing outcomes for eligible families before vs. after FNP was offered; and second, within the same time period but in different LAs, comparing outcomes for eligible families in LAs that did and did not offer FNP.

2. Adjusted analyses

This analysis will be an unmatched comparison, adjusting for maternal characteristics prior to enrolment for each outcome based on causal diagrams and hypothesised influence on effect estimates.

For peer review only - http://bmjopen.bmj.com/site/about/guidelines.xhtml

Sensitivity analyses will determine the strength of unmeasured confounding required to invalidate results.[62 63] To further assess the robustness of findings to the analysis approach and to evaluate any potential differences in results due to the use of real-world data, we will use our cohort to replicate findings observed in the Building Blocks trial. For each analysis strategy, we will derive trial outcomes for a group of families in the administrative data cohort with the same aggregate baseline characteristics as trial participants.

Stratified analyses and interaction terms will be used to investigate effect modification according to the maternal characteristics in Table 2, based on previous evidence suggesting the youngest and most disadvantaged mothers are most likely to benefit from the FNP.

For peer review only

Table 2. Maternal characteristics prior to enrolment (FNP participants) or up to 28 weeks gestation (controls) potentially producing effect modification

- Maternal age at enrolment
- Ethnicity
- Health care use prior to enrolment (unplanned admissions and A&E visits, admissions for drug/alcohol abuse, violence, or self-harm)[58]
- Chronic conditions (including mental health conditions captured in hospital data)[64]
- Educational achievement
- Special educational needs
- Eligibility for Free School Meals
- Out-of-home care
- Area-level deprivation

To identify contextual factors associated with benefitting from FNP (objective 3), we will explore individual and programme characteristics associated with programme engagement, including maternal characteristics prior to enrolment, nurse characteristics, and usual care models in LAs. We will also investigate any differences in FNP effect between LAs, and between our cohort and the corresponding Building Blocks trial population (restricted to trial inclusion criteria), to help generalise trial results.

A detailed statistical plan will be written prior to analyses. Statistical models will take into account clustering within LAs, and also among nurses where appropriate. Data will be analysed in Stata. Study results will be reported in accordance with GUILD, STROBE and RECORD guidelines.[65-67]

Limitations

We do not have individual-level data on primary care, or the number of visits from health visitors that families receive outside of FNP; however, we will seek local aggregate data on usual care models of health visiting and targeted support for teenage mothers over time to examine how these differed across LAs.

For peer review only - http://bmjopen.bmj.com/site/about/guidelines.xhtml

Data on mothers who were offered FNP but declined is not routinely collected. Women who refuse participation may therefore be included in our control group; this may introduce some selection bias, since refusers are likely to differ from FNP accepters in ways that also affect their outcomes. However, propensity score matching sensitivity analyses will restrict controls to women who could not have been offered FNP (either before FNP was offered or in LAs not offering FNP). In the event of remaining unmeasured confounders, grouping refusers with other non-participants may result in an underestimation of FNP effects (if less vulnerable women are more likely to refuse) or overestimation (if more vulnerable women are more likely to refuse). Although individual-level data on refusers is not available, we will obtain data from FNP sites, where available, on the rate and aggregate characteristics of mothers declining participation.

FNP eligibility criteria require enrolment before 28 weeks gestation. Due to a high proportion of missing data on gestational age at first antenatal booking appointment, we will include women who are missing this information as potential controls. 92% of all women present by 20 weeks gestation,[68] therefore any resulting bias in results is likely to be small. However, we will also conduct sensitivity analyses restricting the control group to women presenting up to 28 weeks at booking appointment, to match FNP eligibility criteria.

We estimate at least 90% completeness for linkage of HES and NPD. There may be selection bias in the analyses related to education and social care outcomes due to mothers or children who are not linked being different to those whose records are successfully linked.[51] Nonetheless, data completeness in our cohort is likely to be substantially higher than follow-up rates for medium-term outcomes in most existing trials[15 69 70] (although the Building Blocks trial in England is also using linkage of routine records for follow-up to the child's seventh birthday).[31] Linkage of mother-baby records within HES is high (98%).[38]

Ethics and dissemination

Ethical approval and governance

Support for this study has been obtained from Nottingham Research Ethics Committee (ref 18/EM/0014), from the Department for Education (ref DR190430.02) and the Confidentiality Advisory Group (ref 18/CAG/0013). A Study Steering Committee will oversee the study and ensure the study is conducted to rigorous standards.

Patient and public involvement

In the process of designing our study, we engaged with two groups of mothers (some who had participated in FNP, some who had not). We discussed the use of administrative data for research (including concepts that mothers were not previously familiar with, such as de-identification and data linkage), linkage of health and education data without explicit consent, and the use of these data specifically for evaluating the FNP.

For peer review only - http://bmjopen.bmj.com/site/about/guidelines.xhtml

Workshop participants were supportive of the proposed study and fed-back that linking health and education data for mothers and their children was a good idea. Participants strongly agreed with sharing their data so that services could be improved and future mothers could benefit, and wanted to know how their data had been used to benefit others. Mothers were strongly supportive of taking into account maternal education and area, and wider family support for the FNP, in order to understand whether the programme worked. We incorporated these opinions into our study plan by planning to evaluate whether the effect of FNP differs by area, and by maternal characteristic.

Our Study Steering Committee includes a teenage mother, who will continue to be consulted throughout the study period. Parent participants will help co-produce information on the study for the FNP and institutional websites, and help with interpretation of results and identifying the most appropriate methods of dissemination.

Dissemination

This study will generate policy-relevant findings on the implementation of the FNP in England and likely benefits to maternal and child health, building on evidence from the Building Blocks trial of FNP. By evaluating outcomes for the 31,000 families enrolled in FNP between 2010 and 2017, our study will determine whether there is evidence of benefits for additional outcomes up to age seven and within subgroups (including especially vulnerable young mothers and children). Our findings will directly benefit the Health and Social Care sector by providing LAs, commissioners and policy-makers with detailed and up-to-date evidence to inform the ongoing rollout and targeting of early interventions designed to support young mothers and child development.

Our main output will consist of evidence on the effectiveness of FNP for different groups of families. These results will be disseminated to policy-makers and commissioners in collaboration with the FNP National Unit, and will inform ongoing research into the adaptation of the FNP in England. We will work with parent representatives to co-produce a range of outputs suitable for families participating in FNP. For academic beneficiaries and other researchers, we aim to publish our main findings in high-quality, peer-reviewed journals, as well as present at key conferences. Secondary outputs will include methodological research on the accuracy and reliability of data linkage from health, education and social care sectors, which will inform the use of administrative data by data providers and other researchers.

Acknowledgements

We thank Prof Mike Robling, Dr Rebecca Cannings-John, and Dr Fiona Lugg-Widger from the Building Blocks trial team for their comments and early input into protocol development. We are also grateful to Prof Sally

For peer review only - http://bmjopen.bmj.com/site/about/guidelines.xhtml

Kendall, Andreea Moise and Dr Emma Howarth for their feedback on the study protocol. We are grateful to Dr Lorna Fraser and Dr Kate Walker who provided independent reviews of the study protocol. Study Steering Committee membership: Prof Jane Barlow, Prof Marni Brownell, Dr Lorna Fraser, Dr Emily Petherick, Loretta McGurry and Romy Labrosse.

Author statement

KH is the principal investigator of the study and drafted the original protocol. All authors have contributed to and are responsible for the final design of the study. FC is responsible for dataset preparation and analysis. All authors read and approved the final manuscript.

Funding

This work was supported by the National Institute for Health Research Health Services and Delivery Research (HS&DR) programme (project number 17/99/19). The views and opinions expressed herein are those of the authors and do not necessarily reflect those of the NIHR HS&DR Programme or the Department of Health. Research at UCL Great Ormond Street Institute of Child Health is supported by the NIHR Great Ormond Street Hospital Biomedical Research Centre. RG receives funding from Health Data Research UK (grant No. LOND1), which is funded by the UK Medical Research Council and eight other funders.

Conflicts of interests

We declare we have no conflicts of interest.

Data statement

Data from the Family Nurse Partnership and the Hospital Episode Statistics databases can be obtained from NHS Digital. Data from the National Pupil Database can be obtained from the Department for Education.

List of figures

Figure 1. Data flows diagram

Note: items in italics are identifiers; CIN – Child in Need; CLA – Child Looked After; FNP – Family Nurse Partnership; GP – General Practitioner; HES – Hospital Episode Statistics; NHS – National Health Service; NPD – National Pupil Database; ONS SRS – Office for National Statistics Secure Research Service; PDS – Personal Demographics Service; UCL – University College London

Figure 2. Family Nurse Partnership evaluation outcomes, data sources, and selected maternal characteristics

For peer review only - http://bmjopen.bmj.com/site/about/guidelines.xhtml

References

1. ONS. Births by parents' characteristics 2018. Office for National Statistics; 2019 [Available: https://www.ons.gov.uk/peoplepopulationandcommunity/birthsdeathsandmarriages/livebirths/datasets/birthsbyparentscharacteristics accessed December 2019]
2. Crawford C, Cribb J, Kelly E. Teenage pregnancy in England. 2013 [Available: https://www.ifs.org.uk/caytpubs/caytreport06.pdf accessed December 2019]
3. Wellings K, Palmer MJ, Geary RS, et al. Changes in conceptions in women younger than 18 years and the circumstances of young mothers in England in 2000–12: an observational study. *The Lancet* 2016;388(10044):586-95. doi: https://doi.org/10.1016/S0140-6736(16)30449-4
4. Bellis MA, Hughes K, Leckenby N, et al. National household survey of adverse childhood experiences and their relationship with resilience to health-harming behaviors in England. *BMC Medicine* 2014;12(1):72. doi: 10.1186/1741-7015-12-72
5. Rigsby DC, Macones GA, Driscoll DA. Risk Factors for Rapid Repeat Pregnancy Among Adolescent Mothers: A Review of the Literature. *Journal of Pediatric and Adolescent Gynecology* 1998;11(3):115-26. doi: https://doi.org/10.1016/S1083-3188(98)70130-5
6. Restrepo-Méndez MC, Lawlor DA, Horta BL, et al. The Association of Maternal Age with Birthweight and Gestational Age: A Cross-Cohort Comparison. *Paediatric and Perinatal Epidemiology* 2015;29(1):31-40. doi: 10.1111/ppe.12162
7. Cunnington AJ. What's so bad about teenage pregnancy? *Journal of Family Planning and Reproductive Health Care* 2001;27(1):36-41. doi: 10.1783/147118901101194877
8. Stier DM, Leventhal JM, Berg AT, et al. Are children born to young mothers at increased risk of maltreatment? *Pediatrics* 1993;91(3):642-8.
9. Gonzalez A, MacMillan HL. Preventing child maltreatment: an evidence-based update. *J Postgrad Med* 2008;54(4):280-6. doi: 10.4103/0022-3859.43512
10. Jutte DP, Roos NP, Brownell MD, et al. The Ripples of Adolescent Motherhood: Social, Educational, and Medical Outcomes for Children of Teen and Prior Teen Mothers. *Academic Pediatrics* 2010;10(5):293-301. doi: https://doi.org/10.1016/j.acap.2010.06.008
11. Wiggins M, Oakley A, Sawtell M, et al. Teenage parenthood and social exclusion: a multi-method study - Summary report of findings. London: Social Science Research Unit, Institute of Education; 2005 [Available: https://discovery.ucl.ac.uk/id/eprint/10003007/1/Wiggins2005TeenageParenthood.pdf accessed November 2019]
12. Lawlor D, Shaw M, Johns S. Teenage pregnancy is not a public health problem. *BMJ* 2001;323(7326):1428. doi: 10.1136/bmj.323.7326.1428
13. Olds DL, Hill PL, O'Brien R, et al. Taking preventive intervention to scale: The nurse-family partnership. *Cognitive and Behavioral Practice* 2003;10(4):278-90. doi: https://doi.org/10.1016/S1077-7229(03)80046-9
14. Eckenrode J, Ganzel B, Henderson CR, Jr., et al. Preventing child abuse and neglect with a program of nurse home visitation: the limiting effects of domestic violence. *Jama* 2000;284(11):1385-91. doi: 10.1001/jama.284.11.1385
15. Olds DL, Henderson CR, Jr., Kitzman H. Does prenatal and infancy nurse home visitation have enduring effects on qualities of parental caregiving and child health at 25 to 50 months of life? *Pediatrics* 1994;93(1):89-98.
16. Olds DL, Kitzman H, Hanks C, et al. Effects of nurse home visiting on maternal and child functioning: age-9 follow-up of a randomized trial. *Pediatrics* 2007;120(4):e832-45. doi: 10.1542/peds.2006-2111
17. Kitzman H, Olds DL, Knudtson MD, et al. Prenatal and/or Infancy Nurse Home Visiting and 18-Year Outcomes of a Randomized Trial. *Pediatrics* 2019 doi: 10.1542/peds.2018-3876
18. Olds DL, Robinson J, Pettitt L, et al. Effects of home visits by paraprofessionals and by nurses: age 4 follow-up results of a randomized trial. *Pediatrics* 2004;114(6):1560-8. doi: 10.1542/peds.2004-0961

For peer review only - http://bmjopen.bmj.com/site/about/guidelines.xhtml

19. Mejdoubi J, van den Heijkant SC, van Leerdam FJ, et al. The effect of VoorZorg, the Dutch nurse-family partnership, on child maltreatment and development: a randomized controlled trial. *PLoS One* 2015;10(4):e0120182. doi: 10.1371/journal.pone.0120182
20. Sierau S, Dahne V, Brand T, et al. Effects of Home Visitation on Maternal Competencies, Family Environment, and Child Development: a Randomized Controlled Trial. *Prevention science : the official journal of the Society for Prevention Research* 2016;17(1):40-51. doi: 10.1007/s11121-015-0573-8
21. Segal L, Nguyen H, Gent D, et al. Child protection outcomes of the Australian Nurse Family Partnership Program for Aboriginal infants and their mothers in Central Australia. *PLoS One* 2018;13(12):e0208764. doi: 10.1371/journal.pone.0208764
22. Carabin H, Cowan LD, Beebe LA, et al. Does participation in a nurse visitation programme reduce the frequency of adverse perinatal outcomes in first-time mothers? *Paediatr Perinat Epidemiol* 2005;19(3):194-205. doi: 10.1111/j.1365-3016.2005.00651.x
23. Rubin DM, O'Reilly AL, Luan X, et al. Variation in pregnancy outcomes following statewide implementation of a prenatal home visitation program. *Archives of pediatrics & adolescent medicine* 2011;165(3):198-204. doi: 10.1001/archpediatrics.2010.221
24. Matone M, O'Reilly AL, Luan X, et al. Emergency department visits and hospitalizations for injuries among infants and children following statewide implementation of a home visitation model. *Matern Child Health J* 2012;16(9):1754-61. doi: 10.1007/s10995-011-0921-7
25. Bennett V. Continuing the mandation of the universal five health visiting checks. Public Health England; 2017 [Available: https://publichealthmatters.blog.gov.uk/2017/03/01/continuing-the-mandation-of-the-universal-five-health-visiting-checks/ accessed March 2020]
26. Robling M, Cannings-John R, Channon S, et al. What is usual care for teenagers expecting their first child in England? A process evaluation using key informant mapping and participant survey as part of the Building Blocks randomised controlled trial of specialist home visiting. *BMJ Open* 2018;8(5):e020152. doi: 10.1136/bmjopen-2017-020152
27. Robling M, Bekkers MJ, Bell K, et al. Effectiveness of a nurse-led intensive home-visitation programme for first-time teenage mothers (Building Blocks): a pragmatic randomised controlled trial. *Lancet* 2016;387(10014):146-55. doi: 10.1016/s0140-6736(15)00392-x
28. Olds D. Building evidence to improve maternal and child health. *Lancet* 2016;387(10014):105-7. doi: 10.1016/s0140-6736(15)00476-6
29. Barlow J, Barnes J, Sylva K, et al. Questioning the outcome of the Building Blocks trial. *The Lancet* 2016;387(10028):1615-16. doi: 10.1016/S0140-6736(16)30201-X
30. Fearon RMP. Editorial: Making clinical trials smarter (and more interesting). *Journal of Child Psychology and Psychiatry* 2017;58(2):113-15. doi: 10.1111/jcpp.12696
31. Lugg-Widger FV, Cannings-John R, Channon S, et al. Assessing the medium-term impact of a home-visiting programme on child maltreatment in England: protocol for a routine data linkage study. *BMJ Open* 2017;7(7):e015728. doi: 10.1136/bmjopen-2016-015728
32. Craig P, Dieppe P, Macintyre S, et al. Developing and evaluating complex interventions: the new Medical Research Council guidance. *BMJ* 2008;337:a1655. doi: 10.1136/bmj.a1655
33. Berwick DM. The science of improvement. *Jama* 2008;299(10):1182-4. doi: 10.1001/jama.299.10.1182
34. Kennedy-Martin T, Curtis S, Faries D, et al. A literature review on the representativeness of randomized controlled trial samples and implications for the external validity of trial results. *Trials* 2015;16:495. doi: 10.1186/s13063-015-1023-4
35. Ogilvie D, Cummins S, Petticrew M, et al. Assessing the evaluability of complex public health interventions: five questions for researchers, funders, and policymakers. *The Milbank quarterly* 2011;89(2):206-25. doi: 10.1111/j.1468-0009.2011.00626.x
36. Booth CM, Tannock IF. Randomised controlled trials and population-based observational research: partners in the evolution of medical evidence. *Br J Cancer* 2014;110(3):551-5. doi: 10.1038/bjc.2013.725

For peer review only - http://bmjopen.bmj.com/site/about/guidelines.xhtml

37. Cannings-John R, Lugg-Widger1 F, Lau M, et al. Evaluation of Family Nurse Partnership: methods and process of evaluation. 2020 [Available: https://www.gov.scot/publications/evaluation-family-nurse-partnership-scotland-methods-paper-process-success-linkages/March 2020]
38. Harron K, Gilbert R, Cromwell D, et al. Linking Data for Mothers and Babies in De-Identified Electronic Health Data. *PLOS ONE* 2016;11(10):e0164667. doi: 10.1371/journal.pone.0164667
39. Harron KL, Doidge JC, Knight HE, et al. A guide to evaluating linkage quality for the analysis of linked data. *Int J Epidemiol* 2017;46(5):1699-710. doi: 10.1093/ije/dyx177
40. Herbert A, Wijlaars L, Zylbersztejn A, et al. Data Resource Profile: Hospital Episode Statistics Admitted Patient Care (HES APC). *Int J Epidemiol* 2017;46(4):1093-93i. doi: 10.1093/ije/dyx015
41. ICD. International Classification of Diseases, 10th Revision, Clinical Modification (ICD-10-CM), 2011.
42. Health and Social Care Information Centre. OPCS-4 Classification of Interventions and Procedures.
43. Mc Grath-Lone L, Harron K, Dearden L, et al. Data Resource Profile: Children Looked After Return (CLA). *International journal of epidemiology* 2016;45(3):716-17f. doi: 10.1093/ije/dyw117
44. Department for Education, National Statistics. Characteristics of children in need: 2018-2019. 2019 [Available: https://www.gov.uk/government/statistics/characteristics-of-children-in-need-2018-to-2019 accessed December 2019]
45. Department for Education. Children looked-after by local authorities in England: Guide to the SSDA903 collection 1 April 2019 to 31 March 2020 - Version 1.2. 2019 [Available: https://assets.publishing.service.gov.uk/government/uploads/system/uploads/attachment_data/file/839236/CLA_SSDA903_2019-20_Guide_Version_1.2.pdf accessed December 2019]
46. Mc Grath-Lone L, Dearden L, Nasim B, et al. Changes in first entry to out-of-home care from 1992 to 2012 among children in England. *Child Abuse & Neglect* 2016;51:163-71. doi: https://doi.org/10.1016/j.chiabu.2015.10.020
47. Grath-Lone LM, Woodman J, Gilbert R. Safeguarding children and improving their care in the UK. *The Lancet* 2015;386(10004):1630. doi: 10.1016/S0140-6736(15)00538-3
48. Cattan S. The production of child development in the Family Nurse Partnership. 2017
49. Corbacho B, Bell K, Stamuli E, et al. Cost-effectiveness of the Family Nurse Partnership (FNP) programme in England: Evidence from the building blocks trial. *Journal of Evaluation in Clinical Practice* 2017;23(6):1367-74. doi: 10.1111/jep.12799
50. Health and Social Care Information Centre. Fact Sheet on access methods to trace and use the NHS number on local records. 2014
51. Doidge JC, Harron KL. Reflections on modern methods: linkage error bias. *International Journal of Epidemiology* 2019 doi: 10.1093/ije/dyz203
52. Cannings-John R, Lugg-Widger F, Lau M, et al. Evaluation of Family Nurse Partnership: methods and process of evaluation - Appendix 1: Logic Model. 2020 [Available: https://www.gov.scot/publications/evaluation-family-nurse-partnership-scotland-methods-paper-process-success-linkages/pages/13/ accessed March 2020]
53. Olds D, Henderson CR, Jr., Kitzman H, et al. Effects of prenatal and infancy nurse home visitation on surveillance of child maltreatment. *Pediatrics* 1995;95(3):365-72.
54. WHO. Report of a WHO Technical Consultation on Birth Spacing. Geneva, Switzerland 13-15 June 2005. World Health Organization; 2007 [Available: https://apps.who.int/iris/bitstream/handle/10665/69855/WHO_RHR_07.1_eng.pdf?sequence=1 accessed December 2019]
55. Schnitzer PG, Slusher PL, Kruse RL, et al. Identification of ICD codes suggestive of child maltreatment. *Child Abuse Negl* 2011;35(1):3-17. doi: 10.1016/j.chiabu.2010.06.008
56. Gilbert R, Fluke J, O'Donnell M, et al. Child maltreatment: variation in trends and policies in six developed countries. *Lancet* 2012;379(9817):758-72. doi: 10.1016/s0140-6736(11)61087-8
57. Department for Education, National Statistics. Early years foundation stage profile results in England, 2018. 2018 [Available: https://assets.publishing.service.gov.uk/government/uploads/system/uploads/attachment_data/file/748814/EYFSP_2018_Main_Text.pdf accessed December 2019]

For peer review only - http://bmjopen.bmj.com/site/about/guidelines.xhtml

58. Herbert A, Gilbert R, González-Izquierdo A, et al. Violence, self-harm and drug or alcohol misuse in adolescents admitted to hospitals in England for injury: a retrospective cohort study. *BMJ Open* 2015;5(2):e006079. doi: 10.1136/bmjopen-2014-006079
59. Austin PC. An Introduction to Propensity Score Methods for Reducing the Effects of Confounding in Observational Studies. *Multivariate Behav Res* 2011;46(3):399-424. doi: 10.1080/00273171.2011.568786
60. Blake HA, Leyrat C, Mansfield KE, et al. Propensity scores using missingness pattern information: a practical guide. *arXiv preprint arXiv:190103981* 2019
61. Arpino B, Cannas M. Propensity score matching with clustered data. An application to the estimation of the impact of caesarean section on the Apgar score. *Statistics in medicine* 2016;35(12):2074-91. doi: 10.1002/sim.6880
62. Liu W, Kuramoto SJ, Stuart EA. An introduction to sensitivity analysis for unobserved confounding in nonexperimental prevention research. *Prevention science : the official journal of the Society for Prevention Research* 2013;14(6):570-80. doi: 10.1007/s11121-012-0339-5
63. VanderWeele TJ, Ding P. Sensitivity Analysis in Observational Research: Introducing the E-Value. *Annals of Internal Medicine* 2017;167(4):268-74. doi: 10.7326/m16-2607
64. Hardelid P, Dattani N, Gilbert R. Estimating the prevalence of chronic conditions in children who die in England, Scotland and Wales: a data linkage cohort study. *BMJ Open* 2014;4(8):e005331. doi: 10.1136/bmjopen-2014-005331
65. Gilbert R, Lafferty R, Hagger-Johnson G, et al. GUILD: GUidance for Information about Linking Data sets. *Journal of public health (Oxford, England)* 2018;40(1):191-98. doi: 10.1093/pubmed/fdx037
66. von Elm E, Altman DG, Egger M, et al. Strengthening the Reporting of Observational Studies in Epidemiology (STROBE) statement: guidelines for reporting observational studies. *Bmj* 2007;335(7624):806-8. doi: 10.1136/bmj.39335.541782.AD
67. Benchimol EI, Smeeth L, Guttmann A, et al. The REporting of studies Conducted using Observational Routinely-collected health Data (RECORD) Statement. *PLOS Medicine* 2015;12(10):e1001885. doi: 10.1371/journal.pmed.1001885
68. NHS Digital. Maternity Services Monthly Statistics June 2019, experimental statistics. 2019 [Available: https://digital.nhs.uk/data-and-information/publications/statistical/maternity-services-monthly-statistics/june-2019/analysis accessed March 2020]
69. Olds DL, Kitzman H, Cole R, et al. Effects of nurse home-visiting on maternal life course and child development: age 6 follow-up results of a randomized trial. *Pediatrics* 2004;114(6):1550-9. doi: 10.1542/peds.2004-0962
70. Olds DL, Holmberg JR, Donelan-McCall N, et al. Effects of Home Visits by Paraprofessionals and by Nurses on Children: Follow-up of a Randomized Trial at Ages 6 and 9 Years. *JAMA Pediatrics* 2014;168(2):114-21. doi: 10.1001/jamapediatrics.2013.3817

For peer review only - http://bmjopen.bmj.com/site/about/guidelines.xhtml

For peer review only

For peer review only - http://bmjopen.bmj.com/site/about/guidelines.xhtml

Appendix

Time periods covered by data sources. Shaded boxes indicate cohort member age during study period (blue); look-back period (grey); follow-up (light blue).

	Data source	Year of data																		
		2001	2002	2003	2004	2005	2006	2007	2008	2009	2010	2011	2012	2013	2014	2015	2016	2017	2018	2019
	Births																			
	FNP																			
	HES																			
	NPD																			
	CLA																			
	CIN																			
**Year of birth of cohort member**	1986	15	16	17	18	19	20	21	22	23	24	25	26	27	28	29	30	31	32	33
	1987	14	15	16	17	18	19	20	21	22	23	24	25	26	27	28	29	30	31	32
	1988	13	14	15	16	17	18	19	20	21	22	23	24	25	26	27	28	29	30	31
	1989	12	13	14	15	16	17	18	19	20	21	22	23	24	25	26	27	28	29	30
	1990	11	12	13	14	15	16	17	18	19	20	21	22	23	24	25	26	27	28	29
	1991	10	11	12	13	14	15	16	17	18	19	20	21	22	23	24	25	26	27	28
	1992	9	10	11	12	13	14	15	16	17	18	19	20	21	22	23	24	25	26	27
	1993	8	9	10	11	12	13	14	15	16	17	18	19	20	21	22	23	24	25	26
	1994	7	8	9	10	11	12	13	14	15	16	17	18	19	20	21	22	23	24	25
	1995	6	7	8	9	10	11	12	13	14	15	16	17	18	19	20	21	22	23	24
	1996	5	6	7	8	9	10	11	12	13	14	15	16	17	18	19	20	21	22	23
	1997	4	5	6	7	8	9	10	11	12	13	14	15	16	17	18	19	20	21	22
	1998	3	4	5	6	7	8	9	10	11	12	13	14	15	16	17	18	19	20	21
	1999	2	3	4	5	6	7	8	9	10	11	12	13	14	15	16	17	18	19	20
	2000	1	2	3	4	5	6	7	8	9	10	11	12	13	14	15	16	17	18	19
	2001	0	1	2	3	4	5	6	7	8	9	10	11	12	13	14	15	16	17	18
	2002		0	1	2	3	4	5	6	7	8	9	10	11	12	13	14	15	16	17
	2003			0	1	2	3	4	5	6	7	8	9	10	11	12	13	14	15	16
	2004				0	1	2	3	4	5	6	7	8	9	10	11	12	13	14	15
	2005					0	1	2	3	4	5	6	7	8	9	10	11	12	13	14
	2006						0	1	2	3	4	5	6	7	8	9	10	11	12	13
	2007							0	1	2	3	4	5	6	7	8	9	10	11	12
	2008								0	1	2	3	4	5	6	7	8	9	10	11
	2009									0	1	2	3	4	5	6	7	8	9	10
	2010										0	1	2	3	4	5	6	7	8	9
	2011											0	1	2	3	4	5	6	7	8
	2012												0	1	2	3	4	5	6	7
	2013													0	1	2	3	4	5	6
	2014														0	1	2	3	4	5
	2015															0	1	2	3	4
	2016																0	1	2	3
	2017																	0	1	2

For peer review only - http://bmjopen.bmj.com/site/about/guidelines.xhtml

For peer review only

For peer review only - http://bmjopen.bmj.com/site/about/guidelines.xhtml

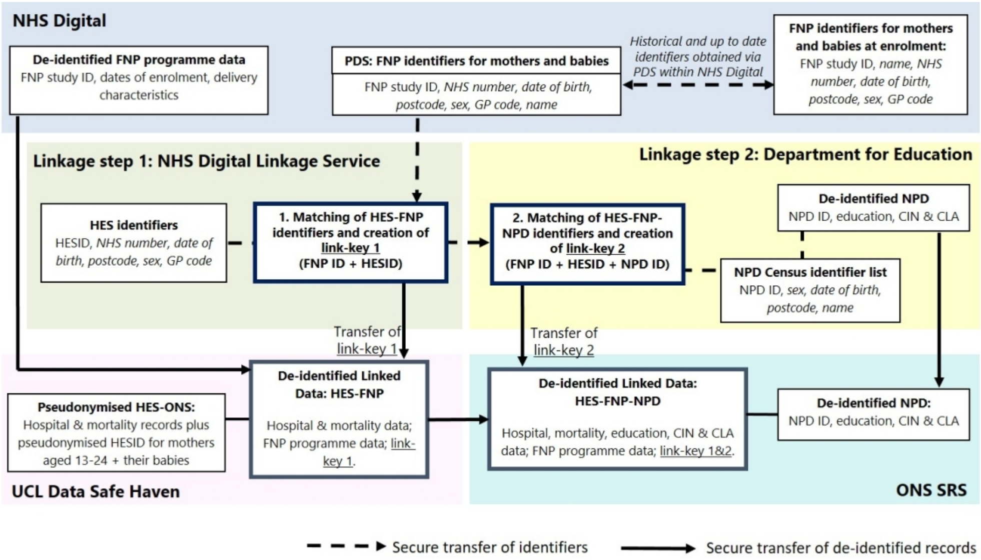

Figure 1. Data flows diagram
Note: items in italics are identifiers; CIN – Child in Need; CLA – Child Looked After; FNP – Family Nurse Partnership; GP – General Practitioner; HES – Hospital Episode Statistics; NHS – National Health Service; NPD – National Pupil Database; ONS SRS – Office for National Statistics Secure Research Service; PDS – Personal Demographics Service; UCL – University College London

124x70mm (300 x 300 DPI)

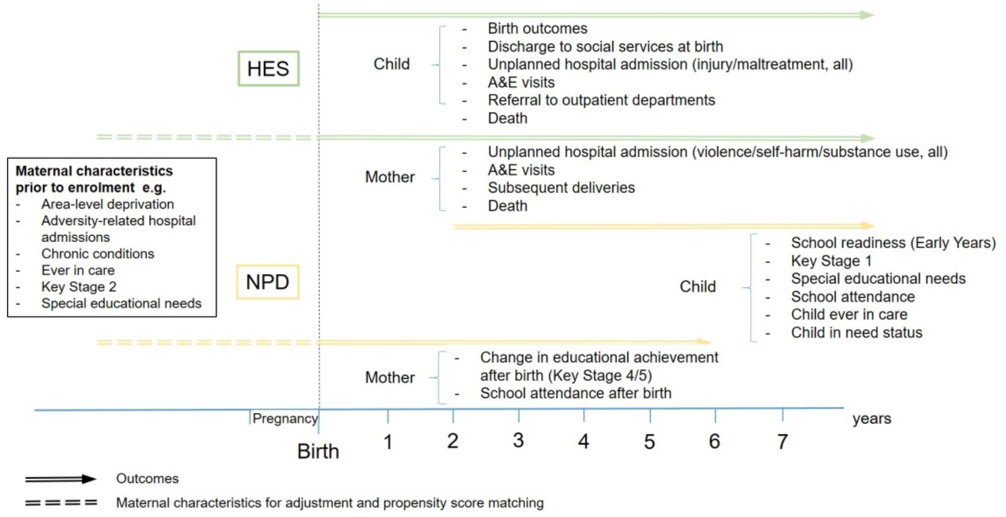

Figure 2. Family Nurse Partnership evaluation outcomes, data sources, and selected maternal characteristics

153x79mm (300 x 300 DPI)

BMJ Open

Evaluating the real-world implementation of the Family Nurse Partnership in England: protocol for a data linkage study

Journal:	*BMJ Open*
Manuscript ID	bmjopen-2020-038530.R1
Article Type:	Protocol
Date Submitted by the Author:	01-Apr-2020
Complete List of Authors:	Cavallaro, Francesca; University College London, Institute of Child Health<br>Gilbert, Ruth; University College London, Institute of Child Health<br>Wijlaars, Linda; University College London, Institute of Child Health<br>Kennedy, Eilis; Tavistock and Portman NHS Foundation Trust<br>Swarbrick, Ailsa; Tavistock and Portman NHS Foundation Trust<br>van der Meulen, Jan; London School of Hygiene & Tropical Medicine, Department of Health Services Research and Policy<br>Harron, Katie; University College London, Institute of Child Health
**Primary Subject Heading**:	Health informatics
Secondary Subject Heading:	Public health
Keywords:	Community child health < PAEDIATRICS, Child protection < PAEDIATRICS, PUBLIC HEALTH, Health informatics < BIOTECHNOLOGY & BIOINFORMATICS

SCHOLARONE™
Manuscripts

I, the Submitting Author has the right to grant and does grant on behalf of all authors of the Work (as defined in the below author licence), an exclusive licence and/or a non-exclusive licence for contributions from authors who are: i) UK Crown employees; ii) where BMJ has agreed a CC-BY licence shall apply, and/or iii) in accordance with the terms applicable for US Federal Government officers or employees acting as part of their official duties; on a worldwide, perpetual, irrevocable, royalty-free basis to BMJ Publishing Group Ltd ("BMJ") its licensees and where the relevant Journal is co-owned by BMJ to the co-owners of the Journal, to publish the Work in this journal and any other BMJ products and to exploit all rights, as set out in our licence.

The Submitting Author accepts and understands that any supply made under these terms is made by BMJ to the Submitting Author unless you are acting as an employee on behalf of your employer or a postgraduate student of an affiliated institution which is paying any applicable article publishing charge ("APC") for Open Access articles. Where the Submitting Author wishes to make the Work available on an Open Access basis (and intends to pay the relevant APC), the terms of reuse of such Open Access shall be governed by a Creative Commons licence – details of these licences and which Creative Commons licence will apply to this Work are set out in our licence referred to above.

Other than as permitted in any relevant BMJ Author's Self Archiving Policies, I confirm this Work has not been accepted for publication elsewhere, is not being considered for publication elsewhere and does not duplicate material already published. I confirm all authors consent to publication of this Work and authorise the granting of this licence.

For peer review only

Evaluating the real-world implementation of the Family Nurse Partnership in England: protocol for a data linkage study

Authors: Francesca L Cavallaro,[1] Ruth Gilbert,[1] Linda Wijlaars,[1] Eilis Kennedy,[2] Ailsa Swarbrick,[2] Jan van der Meulen,[3] Katie L Harron[1]*

[1]UCL GOS Institute of Child Health, London, UK

[2]Tavistock and Portman NHS Foundation Trust, London, UK

[3]Department of Health Services Research and Policy, London School of Hygiene & Tropical Medicine, London, UK

*correspondance: Dr Katie Harron, k.harron@ucl.ac.uk

For peer review only

Abstract

Introduction. Almost 20,000 babies are born to teenage mothers each year in England, with poorer outcomes for mothers and babies than among older mothers. A nurse home visitation programme in the US was found to improve a wide range of outcomes for young mothers and their children. However, a randomised controlled trial in England found no effect on short-term primary outcomes, although cognitive development up to age two showed improvement. Our study will use linked routinely collected health, education and social care data to evaluate the real-world effects of the Family Nurse Partnership (FNP) on child outcomes up to age seven, with a focus on identifying whether the FNP works better for particular groups of families, thereby informing programme targeting and resource allocation.

Methods and analysis. We will construct a retrospective cohort of all women aged 13-24 years giving birth in English NHS hospitals between 2010 and 2017, linking information on mothers and children from FNP programme data, Hospital Episodes Statistics and the National Pupil Database. To assess the effectiveness of FNP, we will compare outcomes for eligible mothers ever and never enrolled in FNP, and their children, using two analysis strategies to adjust for measured confounding: propensity score matching and analyses adjusting for maternal characteristics up to enrolment / 28 weeks gestation. Outcomes of interest include early childhood development, childhood unplanned hospital admissions for injury or maltreatment-related diagnoses, and children in care. Subgroup analyses will determine whether the effect of FNP varied according to maternal characteristics (e.g. age and education).

Ethics and dissemination. The Nottingham Research Ethics Committee approved this study. Mothers participating in FNP were supportive of our planned research. Results will inform policy-makers for targeting home visiting programmes. Methodological findings on the accuracy and reliability of cross-sectoral data linkage will be of interest to researchers.

Keywords: Health informatics, community child health, child protection, public health

Word count: 4,296

For peer review only

Strengths and limitations of this study

- This study will complement results from the Building Blocks trial in England, by evaluating the real-world effect of the Family Nurse Partnership (FNP) on a range of health, educational and social care outcomes up to age 7 years.
- The study cohort comprises joint trajectories for mothers and babies captured in Hospital Episode Statistics (HES; containing >97% births in England and information on subsequent healthcare use), with linked information to FNP programme data and the National Pupil Database (NPD; containing information on children's and mothers' educational attainment and social care).
- Evaluating outcomes for up to 31,000 FNP families and up to 1 million controls will provide statistical power to detect small differences, differences in rare outcomes, and to perform subgroup analyses to determine whether the FNP is more effective in particular groups of families.
- No unique identifiers exist between HES and NPD databases, and linkage may be incomplete.
- Findings will inform the adaptation of the FNP and targeting of support for teenage and young mothers to specific subgroups.

For peer review only - http://bmjopen.bmj.com/site/about/guidelines.xhtml

Introduction

Approximately 3% of babies each year (19,000 in 2018) are born to mothers aged below 20 in England and Wales.[1] Teenage mothers are more likely to experience adversity, be less engaged with education and employment, and have rapid repeat pregnancies compared with older mothers.[2-5] For their children, young maternal age is associated with higher incidence of preterm birth and low birthweight,[6 7] and a greater risk of child maltreatment and associated adverse long-term consequences including poorer physical, social, emotional and cognitive outcomes.[8-10] These adverse maternal and child outcomes of teenage pregnancy, due to social adversity, disruption to education and employment, and child-rearing practices, are of major importance to public health research.[11 12]

The Family Nurse Partnership (FNP) is an intensive home visiting programme that aims to improve outcomes of pregnancies among vulnerable young mothers. Participants receive up to 64 home visits from early pregnancy until the child's second birthday. Specifically, the FNP aims to improve birth outcomes, optimise child health and development, and promote economic self-sufficiency among vulnerable mothers.[13] Three randomised controlled trials in the US found evidence of benefit on a number of maternal and child outcomes, including reductions in child maltreatment, child emergency department visits, and subsequent births, as well as some improvements in child developmental outcomes and longer subsequent birth intervals.[14-18] Effects on hospital visits for injury or maltreatment, children's educational achievements and maternal educational qualifications were less consistent. A trial in the Netherlands found reduced child maltreatment reports by age 2,[19] and a trial of a FNP-based model in Germany reported improved child development among high-risk women only, although no difference in subsequent births within two years.[20] Non-randomised studies have reported reduced child maltreatment,[21] infant death,[22] and subsequent births[23] among FNP participants compared to controls, as well as different patterns of emergency room visits.[24]

Following evidence from the US trials, FNP was introduced in the UK in 2007. The FNP was rolled out via partnerships between local authorities (LA) and healthcare organisations; to date, more than 130 local authorities have implemented the programme. Teenage and young mothers who do not participate in the FNP receive usual care, consisting in comprehensive maternity care and at least five home visits by a health visitor, as well as a varying range of additional services;[25] substantial variation in usual care exists between LAs.[26] In England, the FNP was initially offered to pregnant teenagers aged under 20 years up to 28 weeks gestation, but since 2017 selected sites have expanded to include women up to 24 years with specific vulnerability criteria, and/or have implemented some personalisation of the programme.

Following trials in other countries, the Building Blocks trial randomised 1,618 families in England between 2009 and 2010 and showed no benefit of FNP on primary outcomes up to the child's second birthday

For peer review only - http://bmjopen.bmj.com/site/about/guidelines.xhtml

(smoking in late pregnancy, birthweight, subsequent pregnancy, or child A&E and hospital attendances).[27] However, the interpretation of trial results was debated due to the choice of maternally-reported early childhood development as a secondary outcome, for which a positive effect was observed, and strong support locally for the value of FNP on parent-child relationships.[28-30] Follow-up has been extended up to the child's seventh birthday, with a focus on child maltreatment.[31]

Evaluating complex interventions such as the FNP requires an understanding of the context in which they are delivered as well as the mechanisms of change, in order to investigate and interpret any differences in effects across contexts and populations.[32 33] Indeed, differences in the comparator of usual care may explain differences in results between trials in the US, England, and the Netherlands; these may also vary over time and between LAs in England.[26 28] Furthermore, evidence from the US trials suggests that the youngest, most disadvantaged mothers are likely to benefit most from FNP.[28] Adequately powered subgroup analyses are needed to examine whether some groups of families benefited from FNP more than others. In addition, constrained conditions under which trials are conducted often do not match the complexity of real-world implementation of programmes.[34]

Linkage of existing administrative records provides a cost-efficient means of evaluating services as they are implemented in the real world, by bringing together data from different sectors on a range of outcomes. They also allow for a sufficiently large sample size for subgroup analyses. Our population-based study will use longitudinal observational data to evaluate effects on outcomes of mothers and children up to age seven among eligible families, and generate evidence on the factors that may influence effectiveness and programme engagement (including participant characteristics, setting, provider, and programme delivery).[35 36] Evaluating outcomes for up to 31,000 FNP families and up to 1 million controls will build on the results of the Building Blocks trial by providing increased statistical power to detect smaller differences, differences in rarer outcomes, and subgroup differences for which the Building Blocks trial was underpowered. Study results will inform targeting of services and commissioning by generating evidence on which groups of mothers and their children benefit from the real-world implementation of FNP in England.

Methods and analysis

Study design and population

This study aims to evaluate the real-world, ongoing implementation of the FNP in England on the outcomes of mothers participating in FNP and their children, with the specific objectives of assessing (1) which groups of mothers receive FNP across LAs, (2) the effect of the FNP on maternal and child outcomes, including which families benefit most from FNP, and (3) contextual factors influencing FNP programme effect. It will

For peer review only - http://bmjopen.bmj.com/site/about/guidelines.xhtml

complement results from the Building Blocks trial in England,[27] Building Blocks 2-6 year follow-up,[31] and evaluation of the FNP in Scotland using linked administrative data.[37]

We will create a retrospective cohort of all first-time mothers aged 13-24 years giving birth in England between 1st April 2010 and 31st March 2017 and their children, using individual-level, linked, longitudinal data from routinely collected hospital, education and social care records. Mothers up to 24 years will be included because some FNP sites modified inclusion criteria to include young mothers up to this age. The cohort will be created through linkage of hospital records (Hospital Episode Statistics; HES), education and social care records (National Pupil Database; NPD), and FNP programme data for mothers and their children. Our approach builds on previous linkage of education and health records, and validated methods of linking hospital records for mothers and babies.[38 39] The infrastructure and methodology used to create the linked database will be highly relevant for ongoing evaluation and monitoring of the FNP and other early interventions.

Description of data sources

Hospital records: Hospital Episode Statistics (HES)

Hospital records for mothers aged 13-24 years and their children in England will be extracted from records of births and deliveries in HES. HES is a data warehouse containing details of all hospital admissions (from 1997), outpatient appointments (from 2003) and A&E visits (from 2010) at NHS hospitals in England.[40] HES data have been used extensively in research. In addition to the birth record, we will link information from hospital admissions and A&E visits for mother and child (including up to 11 years before delivery for the mother, see Appendix). Information captured in HES includes administrative data (including admission dates, NHS trust, GP code), demographic information (including age, sex, ethnicity), and clinical information (diagnoses and procedures). A unique 'HESID' is assigned to enable episodes of care for the same individual to be combined. Diagnoses are coded by professional coders in hospitals using ICD-10 codes (International Classification of Disease, version 10); procedures are coded using OPCS-4 codes (Office of Population, Censuses and Surveys Classification of Surgical operations and procedures, version 4).[41 42] Date and cause of death are obtained via linkage between HES and civil registration (deaths) data performed by NHS Digital. Based on previous methodological work, we will link delivery records for mothers and birth records for their children within HES to create a mother-baby HES cohort.[38]

Education and social care records: National Pupil Database (NPD)

The HES cohort of mother-baby pairs will be linked to education and social care records from the Department for Education for both mothers and children in the FNP and control groups (including information before delivery for mothers). Information on assessments, attainment and progression at each

For peer review only - http://bmjopen.bmj.com/site/about/guidelines.xhtml

key stage is available for all pupils in state schools in England, alongside eligibility for free school meals, information about special educational needs and information about absences and exclusions. NPD data, including social care data, have been used extensively in research.[43]

For both mothers and their children in the cohort, we will link HES data to the following NPD databases:

- The yearly School Census (formerly PLASC), the Pupil Referral Unit (PRU) Census, and Alternative Provision – including pupil-level information from 2002 for pupils aged 2-19+ on special educational needs, free school meal eligibility and information about absences and exclusions.
- The Children in Need Census (CINC) – including information on referrals to children's social services, assessments carried out upon these children, and whether the children became the subject of a child protection plan, from 2008.[44]
- The Children Looked After (CLA) return – including information on all looked after children and recent care leavers in England, from 2005.[45 46]

For mothers only, we will link to Key Stages 2, 4 and 5 data, which includes teacher assessments and/or test results in Years 3-6 (ages 7-11), Year 11 (age 16) and Year 12/13 (ages 17-18). For children only, we will link to the Early Years Census and Early Years Foundation Stage Profile (EYFSP), including teacher assessments for three and four year olds (from 2008), and two year olds (from 2014), as well as to Key Stage 1 assessment data (ages 5-6).

A Unique Pupil Number (UPN) is used for linkage of CLA and CINC with the NPD. The UPN is usually assigned at first entry to a maintained school or nursery, typically around the age of four or five. Therefore it is not possible to link the NPD to CLA data for children who are looked after only before they enter nursery/school, or adopted children (who are provided with a new UPN).[47] However, information on out-of-home care available for FNP participants at all ages will enable us to examine the scale of underestimation of out-of-home care before school in the administrative data.

Programme delivery: FNP Information System (FNP IS)

The HES cohort will be linked to the FNP IS to obtain information on participation in FNP. The FNP IS is a bespoke system that supports the implementation of the FNP programme in England, provided by NHS Digital under contract to the FNP National Unit on behalf of Public Health England. Data are reported in real time and are used locally by FNP teams and nationally by the FNP National Unit to monitor programme delivery and support quality improvement.

Data collected in the FNP IS include information from the mother and child collected at enrolment (by 28 weeks gestation at the latest, including mother's age, marital status, living arrangements, education, employment, social care), 36 weeks gestation (including maternal health, alcohol, drugs and smoking), birth (including birthweight and gestational age) and at regular intervals until 24 months after birth (including

For peer review only - http://bmjopen.bmj.com/site/about/guidelines.xhtml

child health and development, social care and other maternal baseline variables). Information on each visit is also collected (including date, length of visit, family nurse seen, and referrals to other services). The FNP IS became functional in 2009, and data quality is reported to be high from 2010 onwards. FNP data have been used in previous research.[48 49]

FNP IS contains maternal and child identifiers at enrolment/birth: name, sex, date of birth, postcode, GP code and NHS number. When mothers graduate from the FNP, pseudonymised data are retained by the FNP National Unit only and identifiers are held solely on secure servers at NHS Digital. Since some identifiers might have changed since enrolment (e.g. mother's name, postcode), identifiers will first be updated using the Personal Demographic Service (PDS) within NHS Digital, so that the most relevant set of identifiers can be used for linkage.[50]

The time span covered by each data source (including look-back, study and follow-up periods) is described in the Appendix.

Data linkage

Data flows are described in Figure 1. Linkage of HES and FNP will be conducted by NHS Digital; linkage with NPD will be performed by the Department for Education. The linkage algorithm for each step (HES-FNP, and HES-FNP-NPD) will be developed in an iterative process, taking into account the completeness of available identifiers (NHS number, GP practice, date of birth, sex, plus name for FNP participants) and building on existing strategies for linkage between HES and NPD. The quality of the linkage will be assessed by estimating the rates and distributions of linkage errors[39 51] according to key maternal and child characteristics. Only de-identified data will be accessed by the research team, and the linked HES-FNP-NPD data will be securely stored on the Office for National Statistics Secure Research Service (ONS SRS).

Outcomes of interest

Study outcomes are described in Figure 2 and Table 1. We selected outcomes for the FNP evaluation based on the FNP logic model,[52] with some caveats outlined below. We will assess the FNP's effect on child abuse and neglect, as measured by unplanned hospital admissions for injury or maltreatment-related diagnoses, child in need status, child looked after status, and death. As the UPN for linking education and social care data is usually assigned at school entry, social care data for children only involved with social care prior to school entry cannot be linked. Therefore, we will only examine child in need and child looked after status after school starting age (4/5). We will also examine the FNP's effect on other child outcomes including uptake and non-attendance for referral to outpatient departments, early years level of development, Key Stage 1 achievement, school attendance, and Special Educational Needs status.

For peer review only - http://bmjopen.bmj.com/site/about/guidelines.xhtml

Some healthcare use outcomes – A&E visits and unplanned hospital admissions for any diagnosis – will be reported as descriptive outcomes, since the direction of effect could be interpreted positively or negatively (FNP participation might reduce the need for emergency care, or alternatively increase appropriate care-seeking). Nonetheless, they represent important outcomes for understanding the effects of FNP on care-seeking behaviour. Similarly, the potential for surveillance bias to distort the effect of early life interventions on child maltreatment has been discussed extensively:[19 27 53] indeed, nurse home visiting results in increased contact with nurses, potentially leading to lower thresholds for reporting among participating families than control group families. This bias in ascertainment of maltreatment may dilute or reverse the association between FNP participation and maltreatment. Conversely, it has also been hypothesised that nurse's closeness to participants may delay reporting of suspected maltreatment.[19]

Maternal outcomes will include unplanned hospital admissions for adversity-related reasons (violence, self-harm, and drug/alcohol abuse) after birth, and death. As with child outcomes, rates of A&E visits and unplanned hospitalisations will be reported as descriptive outcomes. We will also examine the effect of FNP participation on school attendance and achievement after birth, and subsequent pregnancies within 18 months of the first birth ending in live or stillbirths. We will examine subsequent births from a pregnancy within 18 months (rather than 24 months as measured in the Building Blocks trial) because 18 month birth-to-pregnancy intervals are associated with the highest risks of adverse outcomes for women and babies.[54] However, in addition to the outcomes in Table 1, we will validate our findings against the outcomes reported in the Building Blocks trial by examining a range of birth outcomes and subsequent deliveries from pregnancies within 24 months of the index birth.

For peer review only

Table 1. Family Nurse Partnership evaluation outcomes and data sources

Domains	Outcomes	Years after birth	HES	NPD[a]	ONS
**Child outcomes (up to age 7)**					
Child abuse and neglect	Unplanned hospital admissions for any injury or maltreatment-related diagnosis (using ICD10 code lists)[55 56]	0-7	✓		
	Discharge to social services at birth	0	✓		
	Child in care	4/5-7		✓	
	Child in need status	4/5-7		✓	
	Death	0-7			✓
Healthcare use	Unplanned hospital admissions (any diagnoses)	0-7	✓		
	A&E visits (any diagnoses)	0-7	✓		
	Referral to outpatient departments (uptake and non-attendance)	0-7	✓		
Education	Good level of development in early years assessment[57]	5		✓	
	Key Stage 1 assessment	7		✓	
	Special educational needs (SEN) status	5-7		✓	
	School attendance	5-7		✓	
**Maternal outcomes**					
Maternal adversity	A&E visits (any diagnoses)	0-7	✓		
	Unplanned hospital admissions (any diagnoses, and for violence, self-harm, or drug/alcohol abuse)[58]	0-7	✓		
	Death	0-7			✓
Reproductive outcomes	Subsequent deliveries within 18 months of index birth	0-2	✓		
Education	Key Stage 4 and 5 assessment[b]	0-7		✓	
	School attendance after birth[c]	0-7		✓	

[a]Including the School Census, Child in Need Census and Child Looked After databases; [b]Among mothers without Key Stage 4/5 attainment at birth; [c]School attendance information is collected up to age 15

Sample size and power calculation

Exploratory analysis of HES data suggests that approximately 9.5% of children born to first-time teenage mothers are admitted to hospital for injuries by age 6. Assuming a baseline rate of 9.5% for the percentage of children with this outcome up to age 6, a total of 6,798 children (3,399 in each group) would be required to observe a significant relative reduction of 20% in hospital admissions for injuries, with 80% power and a 5% significance level. In our cohort, birth outcomes will be available for around 31,000 FNP births; 24,000 FNP families will have a full 3 years of follow-up and 9,000 will have a full 6 years of follow up. The number of control (never enrolled) families will vary for the propensity score and adjusted analyses; up to one million potential controls (corresponding to total births to women aged 13-24 between April 2010 and March 2017) will be available for these analyses.

Key Stage 4 and 5 assessment outcomes (for ages 14-16 and 16-18, respectively) will be examined among mothers who have not sat these exams before giving birth. We estimate that the sample size for this subgroup will be around 1,800 FNP mothers for Key Stage 4 and 13,000 for Key Stage 5.

For peer review only

For peer review only - http://bmjopen.bmj.com/site/about/guidelines.xhtml

Analysis

We will describe maternal socio-demographic characteristics at the time of pregnancy, previous health and educational characteristics, and pregnancy outcomes for our retrospective cohort of teenage and young mothers (described above).

To determine whether the teenage mothers most in need receive FNP (objective 1), we will identify the proportion of eligible families participating in FNP by LA and over time. Predictors of participation will be derived from HES and NPD data. Since recruiting practices vary locally, we will compare data from high- and low-coverage LAs, to explore whether FNP families in low-coverage LAs are more highly selected than those within LAs offering the programme to more mothers (for example, it may be hypothesised that nurses in some areas may target the most advantaged mothers, or use a first-come-first-served model in others).

To examine the effects of the FNP (objective 2), we will compare outcomes for mothers ever enrolled in FNP, and their children, versus those never enrolled, using two analysis strategies to account for measured confounders related to FNP participation and outcomes. Propensity score matching aims to minimise bias, while adjustment for confounders aims to minimise variance.

1. Propensity score matching

To derive propensity scores, we will construct logistic regression models with FNP participation as the outcome, including as predictors all available maternal characteristics up to 28 weeks gestation (at which point the vast majority of mothers have been enrolled), including looked after status, educational achievement, and pre-pregnancy chronic conditions. Matched groups will be formed by matching FNP participants to eligible non-participants with a similar participation propensity score.[59] Effects will be estimated as the difference in outcomes between matched groups. Multiple imputation will be used to account for missing data where data are missing at random.[60]

The main analysis will restrict matching within the same LA[61] and time period in which FNP was offered within that LA. Secondary analyses aiming to achieve more closely matched groups (with potentially smaller numbers) will match, first, within the same LA but in different time periods, comparing outcomes for eligible families before vs. after FNP was offered; and second, within the same time period but in different LAs, comparing outcomes for eligible families in LAs that did and did not offer FNP.

2. Adjusted analyses

This analysis will be an unmatched comparison, adjusting for maternal characteristics prior to enrolment for each outcome based on causal diagrams and hypothesised influence on effect estimates.

For peer review only - http://bmjopen.bmj.com/site/about/guidelines.xhtml

Sensitivity analyses will determine the strength of unmeasured confounding required to invalidate results.[62 63] To further assess the robustness of findings to the analysis approach and to evaluate any potential differences in results due to the use of real-world data, we will use our cohort to replicate findings observed in the Building Blocks trial. For each analysis strategy, we will derive trial outcomes for a group of families in the administrative data cohort with the same aggregate baseline characteristics as trial participants.

Stratified analyses and interaction terms will be used to investigate effect modification according to the maternal characteristics in Table 2, based on previous evidence suggesting the youngest and most disadvantaged mothers are most likely to benefit from the FNP.

Table 2. Maternal characteristics prior to enrolment (FNP participants) or up to 28 weeks gestation (controls) potentially producing effect modification

- Maternal age at enrolment
- Ethnicity
- Health care use prior to enrolment (unplanned admissions and A&E visits, admissions for drug/alcohol abuse, violence, or self-harm)[58]
- Chronic conditions (including mental health conditions captured in hospital data)[64]
- Educational achievement
- Special educational needs
- Eligibility for Free School Meals
- Out-of-home care
- Area-level deprivation

To identify contextual factors associated with benefitting from FNP (objective 3), we will explore individual and programme characteristics associated with programme engagement, including maternal characteristics prior to enrolment, nurse characteristics, and usual care models in LAs. We will also investigate any differences in FNP effect between LAs, and between our cohort and the corresponding Building Blocks trial population (restricted to trial inclusion criteria), to help generalise trial results.

A detailed statistical plan will be written prior to analyses. Statistical models will take into account clustering within LAs, and also among nurses where appropriate. Data will be analysed in Stata. Study results will be reported in accordance with GUILD, STROBE and RECORD guidelines.[65-67]

Limitations

We do not have individual-level data on primary care, or the number of visits from health visitors that families receive outside of FNP; however, we will seek local aggregate data on usual care models of health visiting and targeted support for teenage mothers over time to examine how these differed across LAs.

For peer review only - http://bmjopen.bmj.com/site/about/guidelines.xhtml

Data on mothers who were offered FNP but declined is not routinely collected. Women who refuse participation may therefore be included in our control group; this may introduce some selection bias, since refusers are likely to differ from FNP accepters in ways that also affect their outcomes. However, propensity score matching sensitivity analyses will restrict controls to women who could not have been offered FNP (either before FNP was offered or in LAs not offering FNP). In the event of remaining unmeasured confounders, grouping refusers with other non-participants may result in an underestimation of FNP effects (if less vulnerable women are more likely to refuse) or overestimation (if more vulnerable women are more likely to refuse). Although individual-level data on refusers is not available, we will obtain data from FNP sites, where available, on the rate and aggregate characteristics of mothers declining participation.

FNP eligibility criteria require enrolment before 28 weeks gestation. Due to a high proportion of missing data on gestational age at first antenatal booking appointment, we will include women who are missing this information as potential controls. 92% of all women present by 20 weeks gestation,[68] therefore any resulting bias in results is likely to be small. However, we will also conduct sensitivity analyses restricting the control group to women presenting up to 28 weeks at booking appointment, to match FNP eligibility criteria.

We estimate at least 90% completeness for linkage of HES and NPD. There may be selection bias in the analyses related to education and social care outcomes due to mothers or children who are not linked being different to those whose records are successfully linked.[51] Nonetheless, data completeness in our cohort is likely to be substantially higher than follow-up rates for medium-term outcomes in most existing trials[15 69 70] (although the Building Blocks trial in England is also using linkage of routine records for follow-up to the child's seventh birthday).[31] Linkage of mother-baby records within HES is high (98%).[38]

Ethics and dissemination

Ethical approval and governance

Support for this study has been obtained from Nottingham Research Ethics Committee (ref 18/EM/0014), from the Department for Education (ref DR190430.02) and the Confidentiality Advisory Group (ref 18/CAG/0013). A Study Steering Committee will oversee the study and ensure the study is conducted to rigorous standards.

Patient and public involvement

In the process of designing our study, we engaged with two groups of mothers (some who had participated in FNP, some who had not). We discussed the use of administrative data for research (including concepts that mothers were not previously familiar with, such as de-identification and data linkage), linkage of health and education data without explicit consent, and the use of these data specifically for evaluating the FNP.

For peer review only - http://bmjopen.bmj.com/site/about/guidelines.xhtml

Workshop participants were supportive of the proposed study and fed-back that linking health and education data for mothers and their children was a good idea. Participants strongly agreed with sharing their data so that services could be improved and future mothers could benefit, and wanted to know how their data had been used to benefit others. Mothers were strongly supportive of taking into account maternal education and area, and wider family support for the FNP, in order to understand whether the programme worked. We incorporated these opinions into our study plan by planning to evaluate whether the effect of FNP differs by area, and by maternal characteristic.

Our Study Steering Committee includes a teenage mother, who will continue to be consulted throughout the study period. Parent participants will help co-produce information on the study for the FNP and institutional websites, and help with interpretation of results and identifying the most appropriate methods of dissemination.

Dissemination

This study will generate policy-relevant findings on the implementation of the FNP in England and likely benefits to maternal and child health, building on evidence from the Building Blocks trial of FNP. By evaluating outcomes for the 31,000 families enrolled in FNP between 2010 and 2017, our study will determine whether there is evidence of benefits for additional outcomes up to age seven and within subgroups (including especially vulnerable young mothers and children). Our findings will directly benefit the Health and Social Care sector by providing LAs, commissioners and policy-makers with detailed and up-to-date evidence to inform the ongoing rollout and targeting of early interventions designed to support young mothers and child development.

Our main output will consist of evidence on the effectiveness of FNP for different groups of families. These results will be disseminated to policy-makers and commissioners in collaboration with the FNP National Unit, and will inform ongoing research into the adaptation of the FNP in England. We will work with parent representatives to co-produce a range of outputs suitable for families participating in FNP. For academic beneficiaries and other researchers, we aim to publish our main findings in high-quality, peer-reviewed journals, as well as present at key conferences. Secondary outputs will include methodological research on the accuracy and reliability of data linkage from health, education and social care sectors, which will inform the use of administrative data by data providers and other researchers.

Acknowledgements

We thank Prof Mike Robling, Dr Rebecca Cannings-John, and Dr Fiona Lugg-Widger from the Building Blocks trial team for their comments and early input into protocol development. We are also grateful to Prof Sally

For peer review only - http://bmjopen.bmj.com/site/about/guidelines.xhtml

Kendall, Andreea Moise and Dr Emma Howarth for their feedback on the study protocol. We are grateful to Dr Lorna Fraser and Dr Kate Walker who provided independent reviews of the study protocol. Study Steering Committee membership: Prof Jane Barlow, Prof Marni Brownell, Dr Lorna Fraser, Dr Emily Petherick, Loretta McGurry and Romy Labrosse.

Author statement

KH is the principal investigator of the study and drafted the original protocol, with input from RG, JvdM and EK. FC and KH produced the first draft of this manuscript; all authors including LW and AS edited the manuscript. All authors have contributed to and are responsible for the final design of the study. FC is responsible for dataset preparation and analysis. All authors read and approved the final manuscript.

Funding

This work was supported by the National Institute for Health Research Health Services and Delivery Research (HS&DR) programme (project number 17/99/19). The views and opinions expressed herein are those of the authors and do not necessarily reflect those of the NIHR HS&DR Programme or the Department of Health. Research at UCL Great Ormond Street Institute of Child Health is supported by the NIHR Great Ormond Street Hospital Biomedical Research Centre. KH is also funded in part by the Wellcome Trust (grant number 212953/Z/18/Z). RG receives funding from Health Data Research UK (grant number LOND1), which is funded by the UK Medical Research Council and eight other funders.

Conflicts of interests

We declare we have no conflicts of interest.

Data statement

Data from the Family Nurse Partnership and the Hospital Episode Statistics databases can be obtained from NHS Digital. Data from the National Pupil Database can be obtained from the Department for Education.

List of figures

Figure 1. Data flows diagram

Note: items in italics are identifiers; CIN – Child in Need; CLA – Child Looked After; FNP – Family Nurse Partnership; GP – General Practitioner; HES – Hospital Episode Statistics; NHS – National Health Service; NPD – National Pupil Database; ONS SRS – Office for National Statistics Secure Research Service; PDS – Personal Demographics Service; UCL – University College London

For peer review only - http://bmjopen.bmj.com/site/about/guidelines.xhtml

For peer review only

Figure 2. Family Nurse Partnership evaluation outcomes, data sources, and selected maternal characteristics

For peer review only

For peer review only - http://bmjopen.bmj.com/site/about/guidelines.xhtml

References

1. ONS. Births by parents' characteristics 2018. 2019. https://www.ons.gov.uk/peoplepopulationandcommunity/birthsdeathsandmarriages/livebirths/datasets/birthsbyparentscharacteristics.
2. Crawford C, Cribb J, Kelly E. Teenage pregnancy in England. 2013. https://www.ifs.org.uk/caytpubs/caytreport06.pdf.
3. Wellings K, Palmer MJ, Geary RS, et al. Changes in conceptions in women younger than 18 years and the circumstances of young mothers in England in 2000–12: an observational study. The Lancet 2016;**388**(10044):586-95.
4. Bellis MA, Hughes K, Leckenby N, et al. National household survey of adverse childhood experiences and their relationship with resilience to health-harming behaviors in England. BMC Medicine 2014;**12**(1):72.
5. Rigsby DC, Macones GA, Driscoll DA. Risk Factors for Rapid Repeat Pregnancy Among Adolescent Mothers: A Review of the Literature. Journal of Pediatric and Adolescent Gynecology 1998;**11**(3):115-26.
6. Restrepo-Méndez MC, Lawlor DA, Horta BL, et al. The Association of Maternal Age with Birthweight and Gestational Age: A Cross-Cohort Comparison. Paediatric and Perinatal Epidemiology 2015;**29**(1):31-40.
7. Cunnington AJ. What's so bad about teenage pregnancy? Journal of Family Planning and Reproductive Health Care 2001;**27**(1):36-41.
8. Stier DM, Leventhal JM, Berg AT, et al. Are children born to young mothers at increased risk of maltreatment? Pediatrics 1993;**91**(3):642-8.
9. Gonzalez A, MacMillan HL. Preventing child maltreatment: an evidence-based update. J Postgrad Med 2008;**54**(4):280-6.
10. Jutte DP, Roos NP, Brownell MD, et al. The Ripples of Adolescent Motherhood: Social, Educational, and Medical Outcomes for Children of Teen and Prior Teen Mothers. Academic Pediatrics 2010;**10**(5):293-301.
11. Wiggins M, Oakley A, Sawtell M, et al. Teenage parenthood and social exclusion: a multi-method study - Summary report of findings. 2005. https://discovery.ucl.ac.uk/id/eprint/10003007/1/Wiggins2005TeenageParenthood.pdf.
12. Lawlor D, Shaw M, Johns S. Teenage pregnancy is not a public health problem. BMJ 2001;**323**(7326):1428.
13. Olds DL, Hill PL, O'Brien R, et al. Taking preventive intervention to scale: The nurse-family partnership. Cognitive and Behavioral Practice 2003;**10**(4):278-90.
14. Eckenrode J, Ganzel B, Henderson CR, Jr., et al. Preventing child abuse and neglect with a program of nurse home visitation: the limiting effects of domestic violence. Jama 2000;**284**(11):1385-91.
15. Olds DL, Henderson CR, Jr., Kitzman H. Does prenatal and infancy nurse home visitation have enduring effects on qualities of parental caregiving and child health at 25 to 50 months of life? Pediatrics 1994;**93**(1):89-98.
16. Olds DL, Kitzman H, Hanks C, et al. Effects of nurse home visiting on maternal and child functioning: age-9 follow-up of a randomized trial. Pediatrics 2007;**120**(4):e832-45.
17. Kitzman H, Olds DL, Knudtson MD, et al. Prenatal and/or Infancy Nurse Home Visiting and 18-Year Outcomes of a Randomized Trial. Pediatrics 2019.
18. Olds DL, Robinson J, Pettitt L, et al. Effects of home visits by paraprofessionals and by nurses: age 4 follow-up results of a randomized trial. Pediatrics 2004;**114**(6):1560-8.
19. Mejdoubi J, van den Heijkant SC, van Leerdam FJ, et al. The effect of VoorZorg, the Dutch nurse-family partnership, on child maltreatment and development: a randomized controlled trial. PLoS One 2015;**10**(4):e0120182.
20. Sierau S, Dahne V, Brand T, et al. Effects of Home Visitation on Maternal Competencies, Family Environment, and Child Development: a Randomized Controlled Trial. Prevention science : the official journal of the Society for Prevention Research 2016;**17**(1):40-51.

For peer review only - http://bmjopen.bmj.com/site/about/guidelines.xhtml

21. Segal L, Nguyen H, Gent D, et al. Child protection outcomes of the Australian Nurse Family Partnership Program for Aboriginal infants and their mothers in Central Australia. PLoS One 2018;**13**(12):e0208764.
22. Carabin H, Cowan LD, Beebe LA, et al. Does participation in a nurse visitation programme reduce the frequency of adverse perinatal outcomes in first-time mothers? Paediatr Perinat Epidemiol 2005;**19**(3):194-205.
23. Rubin DM, O'Reilly AL, Luan X, et al. Variation in pregnancy outcomes following statewide implementation of a prenatal home visitation program. Archives of pediatrics & adolescent medicine 2011;**165**(3):198-204.
24. Matone M, O'Reilly AL, Luan X, et al. Emergency department visits and hospitalizations for injuries among infants and children following statewide implementation of a home visitation model. Matern Child Health J 2012;**16**(9):1754-61.
25. Bennett V. Continuing the mandation of the universal five health visiting checks. 2017. https://publichealthmatters.blog.gov.uk/2017/03/01/continuing-the-mandation-of-the-universal-five-health-visiting-checks/.
26. Robling M, Cannings-John R, Channon S, et al. What is usual care for teenagers expecting their first child in England? A process evaluation using key informant mapping and participant survey as part of the Building Blocks randomised controlled trial of specialist home visiting. BMJ Open 2018;**8**(5):e020152.
27. Robling M, Bekkers MJ, Bell K, et al. Effectiveness of a nurse-led intensive home-visitation programme for first-time teenage mothers (Building Blocks): a pragmatic randomised controlled trial. Lancet 2016;**387**(10014):146-55.
28. Olds D. Building evidence to improve maternal and child health. Lancet 2016;**387**(10014):105-7.
29. Barlow J, Barnes J, Sylva K, et al. Questioning the outcome of the Building Blocks trial. The Lancet 2016;**387**(10028):1615-16.
30. Fearon RMP. Editorial: Making clinical trials smarter (and more interesting). Journal of Child Psychology and Psychiatry 2017;**58**(2):113-15.
31. Lugg-Widger FV, Cannings-John R, Channon S, et al. Assessing the medium-term impact of a home-visiting programme on child maltreatment in England: protocol for a routine data linkage study. BMJ Open 2017;**7**(7):e015728.
32. Craig P, Dieppe P, Macintyre S, et al. Developing and evaluating complex interventions: the new Medical Research Council guidance. BMJ 2008;**337**:a1655.
33. Berwick DM. The science of improvement. Jama 2008;**299**(10):1182-4.
34. Kennedy-Martin T, Curtis S, Faries D, et al. A literature review on the representativeness of randomized controlled trial samples and implications for the external validity of trial results. Trials 2015;**16**:495.
35. Ogilvie D, Cummins S, Petticrew M, et al. Assessing the evaluability of complex public health interventions: five questions for researchers, funders, and policymakers. The Milbank quarterly 2011;**89**(2):206-25.
36. Booth CM, Tannock IF. Randomised controlled trials and population-based observational research: partners in the evolution of medical evidence. Br J Cancer 2014;**110**(3):551-5.
37. Lugg-Widger F, Robling M, Lau M, et al. Evaluation of the effectiveness of the Family Nurse Partnership home visiting programme in first time young mothers in Scotland: a protocol for a natural experiment. International Journal of Population Data Science 2019;**5**(1):12.
38. Harron K, Gilbert R, Cromwell D, et al. Linking Data for Mothers and Babies in De-Identified Electronic Health Data. PLOS ONE 2016;**11**(10):e0164667.
39. Harron KL, Doidge JC, Knight HE, et al. A guide to evaluating linkage quality for the analysis of linked data. Int J Epidemiol 2017;**46**(5):1699-710.
40. Herbert A, Wijlaars L, Zylbersztejn A, et al. Data Resource Profile: Hospital Episode Statistics Admitted Patient Care (HES APC). Int J Epidemiol 2017;**46**(4):1093-93i.
41. ICD. International Classification of Diseases, 10th Revision, Clinical Modification (ICD-10-CM), 2011.
42. Health and Social Care Information Centre. OPCS-4 Classification of Interventions and Procedures.

For peer review only

For peer review only - http://bmjopen.bmj.com/site/about/guidelines.xhtml

43. Mc Grath-Lone L, Harron K, Dearden L, et al. Data Resource Profile: Children Looked After Return (CLA). International journal of epidemiology 2016;**45**(3):716-17f.
44. Department for Education, National Statistics. Characteristics of children in need: 2018-2019. 2019. https://www.gov.uk/government/statistics/characteristics-of-children-in-need-2018-to-2019.
45. Department for Education. Children looked-after by local authorities in England: Guide to the SSDA903 collection 1 April 2019 to 31 March 2020 - Version 1.2. 2019. https://assets.publishing.service.gov.uk/government/uploads/system/uploads/attachment_data/file/839236/CLA_SSDA903_2019-20_Guide_Version_1.2.pdf.
46. Mc Grath-Lone L, Dearden L, Nasim B, et al. Changes in first entry to out-of-home care from 1992 to 2012 among children in England. Child Abuse & Neglect 2016;**51**:163-71.
47. Grath-Lone LM, Woodman J, Gilbert R. Safeguarding children and improving their care in the UK. The Lancet 2015;**386**(10004):1630.
48. Cattan S. The production of child development in the Family Nurse Partnership. 2017.
49. Corbacho B, Bell K, Stamuli E, et al. Cost-effectiveness of the Family Nurse Partnership (FNP) programme in England: Evidence from the building blocks trial. Journal of Evaluation in Clinical Practice 2017;**23**(6):1367-74.
50. Health and Social Care Information Centre. Fact Sheet on access methods to trace and use the NHS number on local records. 2014.
51. Doidge JC, Harron KL. Reflections on modern methods: linkage error bias. International Journal of Epidemiology 2019.
52. Cannings-John R, Lugg-Widger F, Lau M, et al. Evaluation of Family Nurse Partnership: methods and process of evaluation - Appendix 1: Logic Model. 2020. https://www.gov.scot/publications/evaluation-family-nurse-partnership-scotland-methods-paper-process-success-linkages/pages/13/.
53. Olds D, Henderson CR, Jr., Kitzman H, et al. Effects of prenatal and infancy nurse home visitation on surveillance of child maltreatment. Pediatrics 1995;**95**(3):365-72.
54. WHO. Report of a WHO Technical Consultation on Birth Spacing. Geneva, Switzerland 13-15 June 2005. 2007. https://apps.who.int/iris/bitstream/handle/10665/69855/WHO_RHR_07.1_eng.pdf?sequence=1.
55. Schnitzer PG, Slusher PL, Kruse RL, et al. Identification of ICD codes suggestive of child maltreatment. Child Abuse Negl 2011;**35**(1):3-17.
56. Gilbert R, Fluke J, O'Donnell M, et al. Child maltreatment: variation in trends and policies in six developed countries. Lancet 2012;**379**(9817):758-72.
57. Department for Education, National Statistics. Early years foundation stage profile results in England, 2018. 2018. https://assets.publishing.service.gov.uk/government/uploads/system/uploads/attachment_data/file/748814/EYFSP_2018_Main_Text.pdf.
58. Herbert A, Gilbert R, González-Izquierdo A, et al. Violence, self-harm and drug or alcohol misuse in adolescents admitted to hospitals in England for injury: a retrospective cohort study. BMJ Open 2015;**5**(2):e006079.
59. Austin PC. An Introduction to Propensity Score Methods for Reducing the Effects of Confounding in Observational Studies. Multivariate Behav Res 2011;**46**(3):399-424.
60. Blake HA, Leyrat C, Mansfield KE, et al. Propensity scores using missingness pattern information: a practical guide. arXiv preprint arXiv:190103981 2019.
61. Arpino B, Cannas M. Propensity score matching with clustered data. An application to the estimation of the impact of caesarean section on the Apgar score. Statistics in medicine 2016;**35**(12):2074-91.
62. Liu W, Kuramoto SJ, Stuart EA. An introduction to sensitivity analysis for unobserved confounding in nonexperimental prevention research. Prevention science : the official journal of the Society for Prevention Research 2013;**14**(6):570-80.
63. VanderWeele TJ, Ding P. Sensitivity Analysis in Observational Research: Introducing the E-Value. Annals of Internal Medicine 2017;**167**(4):268-74.

For peer review only - http://bmjopen.bmj.com/site/about/guidelines.xhtml

64. Hardelid P, Dattani N, Gilbert R. Estimating the prevalence of chronic conditions in children who die in England, Scotland and Wales: a data linkage cohort study. BMJ Open 2014;**4**(8):e005331.
65. Gilbert R, Lafferty R, Hagger-Johnson G, et al. GUILD: GUidance for Information about Linking Data sets. Journal of public health (Oxford, England) 2018;**40**(1):191-98.
66. von Elm E, Altman DG, Egger M, et al. Strengthening the Reporting of Observational Studies in Epidemiology (STROBE) statement: guidelines for reporting observational studies. Bmj 2007;**335**(7624):806-8.
67. Benchimol EI, Smeeth L, Guttmann A, et al. The REporting of studies Conducted using Observational Routinely-collected health Data (RECORD) Statement. PLOS Medicine 2015;**12**(10):e1001885.
68. NHS Digital. Maternity Services Monthly Statistics June 2019, experimental statistics. 2019. https://digital.nhs.uk/data-and-information/publications/statistical/maternity-services-monthly-statistics/june-2019/analysis.
69. Olds DL, Kitzman H, Cole R, et al. Effects of nurse home-visiting on maternal life course and child development: age 6 follow-up results of a randomized trial. Pediatrics 2004;**114**(6):1550-9.
70. Olds DL, Holmberg JR, Donelan-McCall N, et al. Effects of Home Visits by Paraprofessionals and by Nurses on Children: Follow-up of a Randomized Trial at Ages 6 and 9 Years. JAMA Pediatrics 2014;**168**(2):114-21.

For peer review only

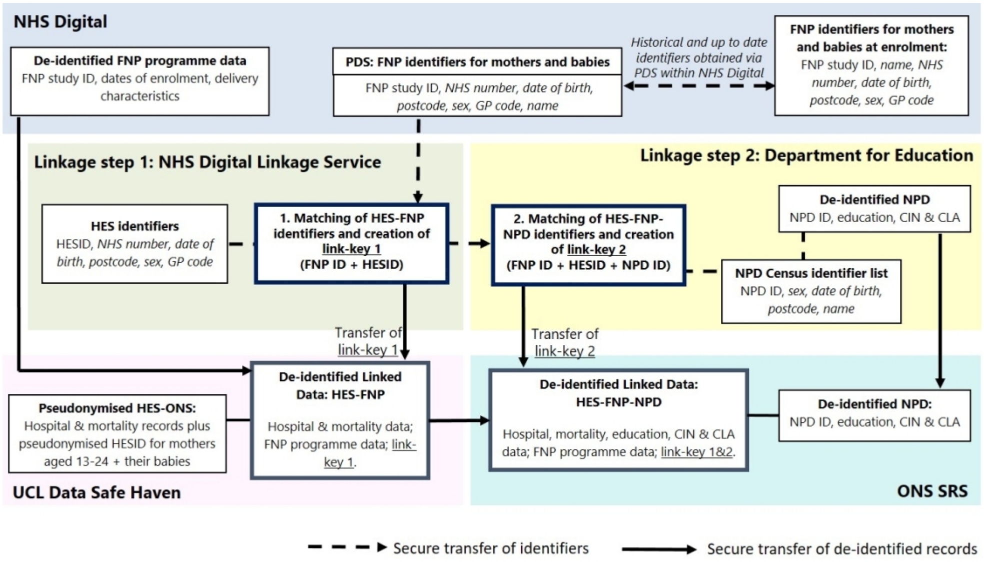

Figure 1. Data flows diagram
Note: items in italics are identifiers; CIN – Child in Need; CLA – Child Looked After; FNP – Family Nurse Partnership; GP – General Practitioner; HES – Hospital Episode Statistics; NHS – National Health Service; NPD – National Pupil Database; ONS SRS – Office for National Statistics Secure Research Service; PDS – Personal Demographics Service; UCL – University College London

124x70mm (300 x 300 DPI)

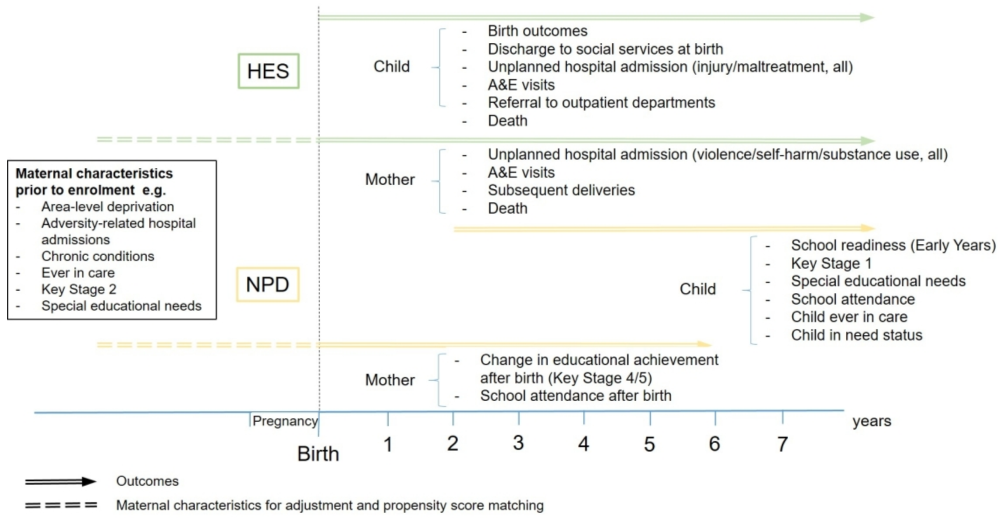

Figure 2. Family Nurse Partnership evaluation outcomes, data sources, and selected maternal characteristics

153x79mm (300 x 300 DPI)

Appendix

Time periods covered by data sources. Shaded boxes indicate cohort member age during study period (blue); look-back period (grey); follow-up (light blue).

	Data source	Year of data 2001	2002	2003	2004	2005	2006	2007	2008	2009	2010	2011	2012	2013	2014	2015	2016	2017	2018	2019
	Births																			
	FNP																			
	HES																			
	NPD																			
	CLA																			
	CIN																			
Year of birth of cohort member	1986	15	16	17	18	19	20	21	22	23	24	25	26	27	28	29	30	31	32	33
	1987	14	15	16	17	18	19	20	21	22	23	24	25	26	27	28	29	30	31	32
	1988	13	14	15	16	17	18	19	20	21	22	23	24	25	26	27	28	29	30	31
	1989	12	13	14	15	16	17	18	19	20	21	22	23	24	25	26	27	28	29	30
	1990	11	12	13	14	15	16	17	18	19	20	21	22	23	24	25	26	27	28	29
	1991	10	11	12	13	14	15	16	17	18	19	20	21	22	23	24	25	26	27	28
	1992	9	10	11	12	13	14	15	16	17	18	19	20	21	22	23	24	25	26	27
	1993	8	9	10	11	12	13	14	15	16	17	18	19	20	21	22	23	24	25	26
	1994	7	8	9	10	11	12	13	14	15	16	17	18	19	20	21	22	23	24	25
	1995	6	7	8	9	10	11	12	13	14	15	16	17	18	19	20	21	22	23	24
	1996	5	6	7	8	9	10	11	12	13	14	15	16	17	18	19	20	21	22	23
	1997	4	5	6	7	8	9	10	11	12	13	14	15	16	17	18	19	20	21	22
	1998	3	4	5	6	7	8	9	10	11	12	13	14	15	16	17	18	19	20	21
	1999	2	3	4	5	6	7	8	9	10	11	12	13	14	15	16	17	18	19	20
	2000	1	2	3	4	5	6	7	8	9	10	11	12	13	14	15	16	17	18	19
	2001	0	1	2	3	4	5	6	7	8	9	10	11	12	13	14	15	16	17	18
	2002		0	1	2	3	4	5	6	7	8	9	10	11	12	13	14	15	16	17
	2003			0	1	2	3	4	5	6	7	8	9	10	11	12	13	14	15	16
	2004				0	1	2	3	4	5	6	7	8	9	10	11	12	13	14	15
	2005					0	1	2	3	4	5	6	7	8	9	10	11	12	13	14
	2006						0	1	2	3	4	5	6	7	8	9	10	11	12	13
	2007							0	1	2	3	4	5	6	7	8	9	10	11	12
	2008								0	1	2	3	4	5	6	7	8	9	10	11
	2009									0	1	2	3	4	5	6	7	8	9	10
	2010										0	1	2	3	4	5	6	7	8	9
	2011											0	1	2	3	4	5	6	7	8
	2012												0	1	2	3	4	5	6	7
	2013													0	1	2	3	4	5	6
	2014														0	1	2	3	4	5
	2015															0	1	2	3	4
	2016																0	1	2	3
	2017																	0	1	2
