# Supplementary material for: Evaluating the real-world implementation of the Family Nurse Partnership in England: protocol for a data linkage study
Source: BMJ Open. 2020 May 18;10(5):e038530. doi: 10.1136/bmjopen-2020-038530 (PMC7239518; doi:10.1136/bmjopen-2020-038530)
Supplement: Reviewer comments [file bmjopen-2020-038530.reviewer_comments.pdf]

PEER REVIEW HISTORY

BMJ Open publishes all reviews undertaken for accepted manuscripts. Reviewers are asked to complete a checklist review form (**http://bmjopen.bmj.com/site/about/resources/checklist.pdf**) and are provided with free text boxes to elaborate on their assessment. These free text comments are reproduced below.

ARTICLE DETAILS

**TITLE (PROVISIONAL)**	Evaluating the real-world implementation of the Family Nurse Partnership in England: protocol for a data linkage study
**AUTHORS**	Cavallaro, Francesca; Gilbert, Ruth; Wijlaars, Linda; Kennedy, Eilis; Swarbrick, Ailsa; van der Meulen, Jan; Harron, Katie

This article was not externally reviewed at BMJ Open. Protocols that have been independently assessed prior to submission to BMJ Open are usually fast-tracked to publication on the grounds that further substantial changes will not be possible. This independent assessment will usually be external, independent review for both a substantial grant award from a non-commercial or government funder and ethics approval.
